# *Christensenella intestinihominis* MNO-863 improve obesity and related metabolic disorders via SCFAs-induced GLP-1 hormone secretion

**DOI:** 10.3389/fnut.2025.1668786

**Published:** 2025-11-20

**Authors:** Ping Kong, Yibo Xian, Canshan Lao, Baojia Huang, Dongya Zhang, Lihong Tai, Yingying Zhao, Zilun Pu, Zhou Lan, Chenchen Zhang, Zhenzhen Liu, Chen Xiao, Guozhen Zhao, Ruijuan Zhu, Yajun Liang, Chuan-Sheng Lin, Jing-han Lin, Jing-zu Sun, Tao Wang, Hong-Wei Liu, Xianzhi Jiang

**Affiliations:** 1 Moon (Guangzhou) Biotech Co. Ltd., Guangzhou, Guangdong, China; 2 State Key Laboratory of Microbial Diversity and Innovative Utilization, Institute of Microbiology, Chinese Academy of Sciences, Beijing, China

**Keywords:** anti-obesity, metabolic disorders, *Christensenella intestinihominis*, SCFAs, GLP-1, probiotic, insulin resistance, NASH

## Abstract

The intestinal microbiota has been demonstrating a strong correlation with the etiology and progression of obesity and metabolic disorders, thus presenting a novel approach to addressing this issue. In this study, we screened and revealed the anti-obesity efficacy of the viable *Christensenella intestinihominis* (*C. intestinihominis*) MNO-863 in diet-induced obese mouse models. MNO-863 reduced body weight by 10% from baseline and over 15% compared to high-fat control in the dose-dependent manner. It also ameliorated obesity-related metabolic indices including hyperlipidemia, hyperglycemia, glucose and insulin resistance, and non-alcoholic steatohepatitis (NASH). The anti-obesity efficacy of MNO-863 monotherapy is comparable to that of Liraglutide (GLP-1 analogue), and the combination of MNO-863 and Liraglutide has potential synergistic anti-obesity therapeutic effect. Treatment with MNO-863 significantly raised the levels of intestinal hormones, such as glucagon-like peptide-1 (GLP-1) and peptide YY (PYY), and concurrently increased the abundance of short-chain fatty acids (SCFAs) producing bacteria, resulting in higher colonic concentrations of propionate. These changes are correlative with previous observations suggesting that propionate–G-protein coupled receptor 43 (GPR43) interaction may contribute to GLP-1 and PYY release; causality remains to be established. A 28-day oral toxicity study in Sprague Dawley (SD) rats showed that MNO-863 Fermental Powder at doses up to 1.2 × 10^12^ colony-forming unit (CFU)/animal/day caused no observed adverse effects. As a second-generation probiotic, MNO-863 is expected to provide a new, safer drug option for patients with obesity and related complications.

## Introduction

The global rise in obesity and associated metabolic conditions has become a major health concern, significantly increasing the risk of severe illnesses, such as heart disease, various cancers, diabetes, and osteoarthritis (1, 2). Current treatments for obesity and related metabolic disorders have limitations (3). Diet control and exercise therapy require long-term commitment and high self-discipline, leading to poor compliance and common weight rebound (4). Approved weight-loss drugs are few and often cause adverse reactions like gastrointestinal discomfort and increase cardiovascular risk, limiting their use (4). Surgical treatment is effective for weight loss and metabolic improvement, but surgery risks, postoperative complications, and high costs deter many patients (5). Therefore, developing new, efficient, and safe treatment strategies are urgently needed.

The human gut microbiome significantly influences health outcomes, including metabolic health, infection susceptibility, and immune diseases (6, 7). Numerous studies indicate that the gut flora of obese individuals differs markedly from those of normal weight individuals (8). Fecal microbiota transplantation and specific bacterial strains have been demonstrated to improve metabolic conditions by influencing sugar and lipid metabolism, energy homeostasis, inflammation, and gut barrier function (9). Importantly, gut flora metabolites like SCFAs (10), succinate (11), bile acids (12), indoles (13), and branched-chain amino acids (8) are crucial for regulating energy balance, improving glucose and lipid metabolism, and maintaining metabolic homeostasis. SCFAs, primarily produced through the digestion of dietary fiber and indigestible carbohydrates by anaerobic gut bacteria, include acetic acid, propionic acid, and butyric acid, each with unique physiological functions (10). They act on G protein-coupled receptors (GPCRs) on intestinal endocrine cells, such as GPR41 and GPR43, promoting the secretion of GLP-1 and PYY (14). GLP-1 and PYY can stimulate insulin secretion, enhance insulin sensitivity, transmit satiety signals to the central nervous system, and regulate energy balance (14, 15). SCFAs can also directly signal to the adipose tissue, regulating energy utilization in adipose tissue and liver, increasing fat breakdown and reducing liver fat accumulation, thereby improving obesity in mice (16).

Advances in multi-omics sequencing have identified unique microbial taxonomic and functional traits linked to metabolic disorders, presenting potential therapeutic targets (8). A significant variability in the abundance of intestinal bacteria in the family of *Christensenella* was indicated among individuals, with lower levels in obese individuals (17). Cohort data analysis has illuminated the correlation between *Christensenellaceae* and metabolic diseases, as well as its potential role in ameliorating these conditions (17, 18). Recently, eighty-seven strains of *Christensenellaceae* encompassing seven new species from eight genera have been described (19). Among these strains, *Christensenella minuta* (*C. minuta*) was well studied due to its beneficial effects on obesity and its related metabolic disorders (20). Recent studies have shown that *C. minuta* modulates host metabolism by converting host bile acids into 3-O-acylated cholic acids (e.g., 3-O-acetyl/propionyl/butyryl-CA) in the gut, which act as FXR antagonists (21). *C. intestinihominis* is a new species of *Christensenella* that was reported in 2021, and there is no direct evidence of its weight-loss effect (19). In this study, we first demonstrated the therapeutic properties of *C. intestinihominis* MNO-863, a strain selected from a microbial library containing over 208 strains from six species (*Christensenella hongkongensis*, *C. intestinihominis*, *Christensenella massiliensis*, *C. minuta*, *Christensenella timonensis* and *Christensenella* sp.). Viable MNO-863 ameliorated obesity-related metabolic indices and the combination of MNO-863 with Liraglutide also showed potential synergistic anti-obesity effects. Furthermore, the action mechanisms of MNO-863 were investigated by analysis of gut microbiome and metabolome. The therapeutic effects of MNO-863 are accompanied by an enhancement of the abundance of SCFA-producing intestinal bacteria and a concomitant rise in colonic propionate. Consistent with prior reports that propionate can interact with GPR43 and GPR41 in enteroendocrine cells (14), we observed up-regulation of both GPR43 and GPR41 alongside increased GLP-1 and PYY levels. Whether this association reflects a causal axis requiring GPR43 remains to be directly tested. Therefore, *C. intestinihominis* MNO-863, a second-generation probiotic, has the potential to be a new live biotherapeutic product (LBP) for anti-obesity treatment.

## Materials and methods

### Bacterial isolation and cultivation

*Christensenella* spp. strains, including MNO-863, used in this study were isolated from healthy human fecal samples in the laboratory, determined by their full 16S rRNA and confirmed by their whole genomic sequence. *C. intestinihominis* MNO-863 was deposited under GDMCC No: 61117 in Guangdong Microbial Culture Collection Center (GDMCC) at Guangdong Institute of Microbiology. They were cultured in self-optimized MM01 liquid medium (peptone, 15 g; glucose, 20 g; yeast extract, 15 g; cysteine, 1 g; sodium acetate, 5 g; sodium citrate, 4 g; dipotassium phosphate, 2 g; magnesium sulfate, 0.1 g; manganese sulfate, 0.05 g; Tween 80, 1 g contained per liter; pH 6.3–6.5) and grown anaerobically under an atmosphere of 10% CO2, 10% H2 and 80% N2 at 37 °C for 48 h. All the reagents were vented in an anaerobic atmosphere for at least 24 h prior to use. MNO-863 culture was sterilized by autoclave at 121 °C for 20 min to obtain Heat-Killed MNO-863, followed by immediate refrigeration at −80 °C until further use. In addition, MNO-863 culture was centrifuged and re-suspended in protective agent and then freeze-dried for 48 h in a vacuum freeze-dryer to obtain MNO-863 Fermental Powder. Amplification of the bacteria 16S rRNA genes was performed using the primers 27F (5'-AGAGTTTGATCCTGGCTCAG-3') and 1492R (5'-GGTTACCTTGTTACGACTT-3'). Sequencing data of the bacterial 16S rRNA genes is available in NCBI GenBank (Accession: PX470668).

### Experimental animals

Six to eight weeks old male C57BL/6J (GemPharmatech Inc.) were maintained in a specific pathogen free environment. Experiment 1 ~ 4 were carried out according to protocols approved by the Institutional Animal Care and Use Committee of Moon Biotech Co., Ltd., in compliance with the Guide for the Care and Use of Laboratory Animals. The animals were housed in a controlled environment (24 ± 1 °C, 12-h daylight cycle, lights off at 18:00) with ad libitum access to food and water.

**Figure 1:**
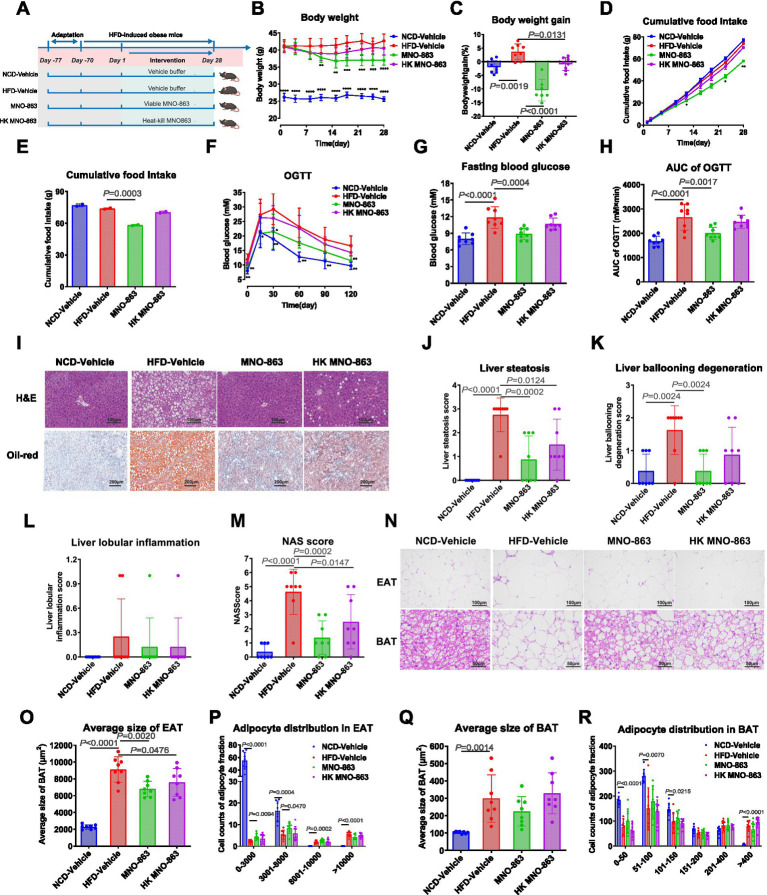
Viable MNO-863 administration improved obesity-related metabolic indicators in HFD-induced obese mice. **(A)** Schematic diagram of Experiment 1 (NCD-Vehicle, *n* = 8; HFD-Vehicle, *n* = 8; MNO-863, *n* = 8; HK MNO-863, *n* = 8). **(B)** Curve graph of body weight. **(C)** Body weight gain (%) at on Day28. **(D)** Curve graph of cumulative food intake. **(E)** Cumulative food intake during intervention. **(F)** OGTT curve. **(G)** Fasting blood glucose of OGTT. **(H)** Area under curve of OGTT. **(I)** Representative H&E and oil-red staining photographs of livers with 200-fold field, scale: 100 μm. **(J)** Liver steatosis. **(K)** Liver ballooning degeneration. **(L)** Liver lobular inflammations. **(M)** NAFLD Activity Score (NAS). **(N)** Representative H&E staining photographs of EAT and BAT, with 400-fold field, scale: 100 μm. **(O)** Average adipocyte size of EAT. **(P)** Number of EAT cells by different size. **(Q)** Average adipocyte size of BAT. **(R)** Number of BAT cells by different size. Data are presented as mean ± SD. Statistical analysis was performed by two-way ANOVA for the line chart or one-way ANOVA for the bar chart combined with Dunnett’s multiple comparisons test, ns: not significant, not showed; ^*^p < 0.05, ^**^p < 0.01, ^***^p < 0.001, ^****^p < 0.0001.

For (i) Experiment 1: After 1 week of acclimatization, male C57BL/6J mice were fed either a NCD (normal control diet; D12450B, Research Diet) or an HFD (high-fat diet, D12492, Research Diet) for 10 weeks to build an obesity model. NCD- and HFD-fed mice were randomly grouped according to body weight using a random ranking table method (22). Then, we treated with NCD mice with vehicle buffer (NCD-Vehicle), and HFD-induced obese mice with vehicle buffer (HFD-Vehicle), viable MNO-863 (MNO-863, 1.2 × 10^11^ CFU/day) or Heat-Killed MNO-863 (HK MNO-863, 0 CFU/day) by oral gavage for a period of 4 weeks (Figure 1A).

**Figure 2:**
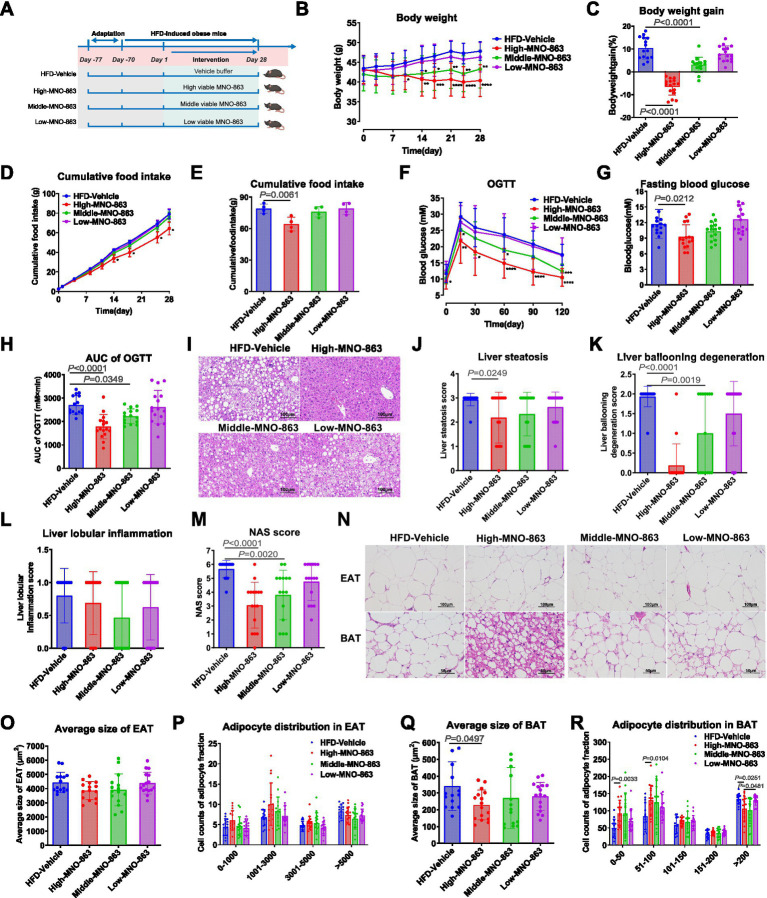
MNO-863 improved obesity-related symptoms in HFD-induced obese mice in the dose-dependent manner. **(A)** Schematic diagram of Experiment 2 (HFD-Vehicle, *n* = 16; High-MNO-863, *n* = 16; Middle-MNO-863, *n* = 16; Low-MNO-863, *n* = 16). **(B)** Curve graph of body weight. **(C)** Body weight gain (%) at on Day28. **(D)** Curve graph of cumulative food intake. **(E)** Cumulative food intake during intervention. **(F)** OGTT curve. **(G)** Fasting blood glucose of OGTT. **(H)** area under curve of OGTT. **(I)** Representative H&E staining photographs of livers, with 200-fold field, scale: 100 μm. **(J)** Liver steatosis. **(K)** Liver ballooning degeneration. **(L)** Liver lobular inflammations. **(M)** NAS Score. **(N)** Representative H&E staining photograph of EAT and BAT, with 400-fold field, scale: 100 μm. **(O)** Average adipocyte size of EAT. **(P)** Number of EAT cells by different size. **(Q)** Average adipocyte size of BAT. **(R)** Number of BAT cells by different size. Data are presented as mean ± SD. Statistical analysis was performed by two-way ANOVA for the line chart or one-way ANOVA for the bar chart combined with Dunnett’s multiple comparisons test, ns: not significant, not showed; ^*^p < 0.05, ^**^p < 0.01, ^***^p < 0.001, ^****^p < 0.0001.

For (ii) Experiment 2: After 1 week of acclimatization, male C57BL/6J mice were fed an HFD for 10 weeks to build an obesity model. HFD-fed mice were randomly grouped according to body weight using a random ranking table method. Then, we treated HFD-mice with vehicle buffer (HFD-Vehicle), High dose (High-MNO-863, 1.2 × 10^11^ CFU/day), Middle dose (Middle-MNO-863, 2.4 × 10^10^ CFU/day), Low dose (Low-MNO-863, 2.4 × 10^9^ CFU/day) by oral gavage for a period of 4 weeks (Figure 2A).

**Figure 3:**
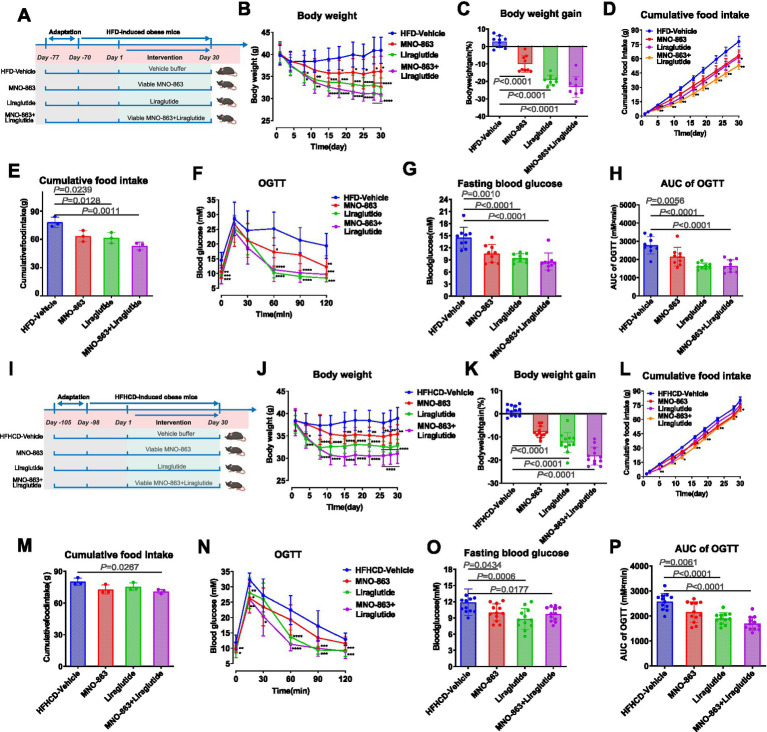
Viable MNO-863 and MNO-863 + Liraglutide administration improved obesity-related metabolic indicators in HFD- and HFHCD-induced Obese Mice. **(A)** Schematic diagram of Experiment 3 (HFD-Vehicle, *n* = 9; MNO-863, *n* = 9; Liraglutide, *n* = 9; Liraglutide+MNO-863, *n* = 9). **(B)** Curve graph of body weight. **(C)** Body weight gain (%) at on Day30. **(D)** Curve graph of cumulative food intake. **(E)** Cumulative food intake during intervention. **(F)** OGTT curve. **(G)** Fasting blood glucose of OGTT. **(H)** Area under curve of OGTT. **(I)** Schematic diagram of Experiment 4 (HFHCD-Vehicle, *n* = 12; MNO-863, *n* = 12; Liraglutide, *n* = 12; Liraglutide+MNO-863, *n* = 12). **(J)** Curve graph of body weight. **(K)** Body weight gain (%) at on Day30. **(L)** Curve graph of cumulative food intake. **(M)** Cumulative food intake during intervention. **(N)** OGTT curve. **(O)** Fasting blood glucose of OGTT. **(P)** Area under curve of OGTT. Data are presented as Mean ± SD. Statistical analysis was performed by two-way ANOVA for the line chart or one-way ANOVA for the bar chart combined with Dunnett’s multiple comparisons test. ns: not significant, not showed; ^*^p < 0.05, ^**^p < 0.01, ^***^p < 0.001, ^****^p < 0.0001.

For (iii) Experiment 3: After 1 week of acclimatization, male C57BL/6 J mice were fed an HFD for 10 weeks to build an obesity model. Then, we treated HFD-mice with vehicle buffer (HFD-Vehicle), Viable MNO-863 (MNO-863, 1.2 × 10^11^ CFU/day), positive control (Liraglutide) or combination (MNO-863 + Liraglutide) for a period of 30 days (Figure 3A).

For (iiii) Experiment 4: After 1 week of acclimatization, male C57BL/6J mice were fed an HFHCD (High-fat, High-fructose and High-cholesterol Diet, D09100310, Research Diet) for 14 weeks to build an obesity model. Then, we treated HFHCD-mice with vehicle buffer (HFHCD-Vehicle), Viable MNO-863 (MNO-863, 1.2 × 10^11^ CFU/day), positive control (Liraglutide, 100 μg/kg/day) or combination (MNO-863 + Liraglutide) for a period of 30 days (Figure 3I).

The volume of test article and vehicle buffer with gavage was 200 μL/animal/time, twice daily. The dose of Liraglutide with administered subcutaneously was 100 μg/kg/day, once daily. According to experimental procedure, body weight and 24-h food intake were measured twice weekly. Glucose tolerance tests had been previously described (23). After fasting overnight at the end of the experiment, mice were anesthetized using an inhalation anesthesia system (Shanghai Yuyan Instruments Co., Ltd., CL-1000-S4). Anesthesia was induced with 4–5% isoflurane (Jinan Ante Biochemical Pharmaceutical Co., Ltd., R510-22-10) delivered in oxygen at a flow rate of 200–400 mL/min within an induction chamber. After the loss of righting reflex, mice were transferred to a nose cone, and surgical anesthesia was maintained with 1.5–2.5% isoflurane. Blood sampling from the orbital venous plexus was then performed under terminal anesthesia, whereupon the mice were immediately euthanized by cervical dislocation. Serum chemistry parameters, such as Cholesterol (CHO), Triglycerides (TG), Low-Density Lipoprotein (LDL), High-Density Lipoprotein (HDL), Alanine Aminotransferase (ALT), Aspartate Aminotransferase (AST) were tested by Wuhan Servicebio Technology Co., Ltd. using automatic biochemical analyzer.

### Pathology examination of liver and adipose tissue

Liver and adipose tissue were fixed in 4% paraformaldehyde and subjected to H&E or red-oil staining, followed by pathology examination by Wuhan Servicebio Technology Co., Ltd. The degree of NAFLD (Non-alcoholic Fatty Liver Disease) severity was determined using inflammation, steatosis, and ballooning scores and the NAFLD activity score (NAS) (24). Semi-quantification results of mean size of adipocytes and the number of different adipocyte size intervals were generated and analyzed using Image J (23).

### Analysis of plasma hormones and obesity-related factors

Plasma hormones and obesity-related factors were analyzed by Luminex detection technology combined with MILLIPLEX® Mouse Metabolic Hormone Expanded Panel (MMHE-44 K, 15-plex, Merck-Millipore, USA) and LXSAMSM-23 and LXSAMSM-01 mouse magnetic bead multi-factor assay kits (LXSAMSM-23, 01, R&D Systems, Minneapolis, MN, USA), which was performed by Shanghai Global Biotechnology Co., LTD. (25).

### Immunohistochemistry

The ileum tissues of the mice were collected, fixed with 4% neutral paraformaldehyde. Paraffin sections were sequentially placed in xylene, gradient ethanol (100, 95, 80, 70%), and finally washed with distilled water, followed by antigen repair, endogenous peroxidase blocking and serum blocking (26). The ileum sections were individually incubated with specific antibodies against GPR41 (Proteintech, 66,811-1-IG) or GPR43 (Proteintech, 19,952-1-AQ) and HRP-conjugated secondary antibodies (DAKO, K5007). The samples were then subjected to DAB color (Diaminobenzidine Color Development Kit, DAKO, K5007) development, restraining of nuclei, dehydration sealing and examined under a fluorescent white light microscope (NIKON ECLIPSE C1) (27). Finally, the images were analyzed with Lndica Labs (Halo 3.4.2986).

### Assessment of bacterial-stimulated GLP-1 secretion *in vitro*

NCI-H716 human intestinal L-cells (Wuhan Pricella Biotechnology Co., Ltd) were cultured in DMEM medium (Gibco) supplemented with 10% fetal bovine serum (FBS, Gibco) and penicillin–streptomycin (Gibco) at 37 °C and 5% CO2. For the assay, cells were seeded into 96-well plates that had been coated with 100 μL of a matrix gel (Corning) and incubated at room temperature for 2 h prior to cell plating. The cells were plated at a density of 1 × 10^5^ cells per well and cultured for 48 h. Following this, the cells were washed twice with HBSS Buffer (28). MNO-863 cell-free supernatants (CFS) were then added at volume ratios of 5% or 10%, and the cells were incubated for an additional 2 h. After incubation, the supernatants were collected for GLP-1 assay, which was quantitatively measured using the GLP-1 enzyme-linked immunosorbent assay (ELISA) kit (Millipore, cat.no EGLP-35K) according to the manufacturer’s instructions.

### Metabolomics data analysis

Metabolomic analysis was carried out by Metabo-Profile Biotechonology (Shanghai) Co. Ltd. Metabolites from the cecal content and liver tissue were extracted using chilled 80% methanol and 0.1% formic acid, followed by purification with 60% methanol LC–MS grade water before being analyzed by an LC–MS/MS system. The UHPLC–MS/MS analysis was conducted on a Vanquish UHPLC system (ThermoFisher, Germany) interfaced with an Orbitrap Q Exactive HF mass spectrometer (ThermoFisher, Germany). Raw data were processed with Compound Discoverer (V3.1) for metabolite identification (29, 76). The normalized data were utilized to deduce molecular formulas, and peak matches were referenced against the mzCloud, mzVault, and MassList databases. Statistical analysis was performed using R and Python scripts. To deduce the variations between samples, we conducted principal components analysis (PCA) to show the differences in metabolome among samples. The differential analysis of metabolite was conducted by linear discriminant analysis effect size (LEfSe, LDA > 2, *p* < 0.05) (30). For differential analysis at the metabolite class level, either the Wilcoxon rank-sum test or Student’s t-test was applied, based on the normality of the data distribution. Metabolite annotations were based on the KEGG, HMDB, and LIPIDMaps databases. Raw data is available in MetaboLights (Accession: MTBLS13079).

### Cecal metagenomic analysis

Metagenomic sequencing of cecal content samples was performed by Novogene (Beijing, China), and data analysis was performed by the Moon (Guangzhou) Biotech Co., Ltd. Genomic DNA extraction, library establishment and sequencing process of cecal contents samples had been previously described (31). After library quality inspection was qualified, Illumina PE150 sequencing was performed to obtain raw sequencing data. Raw data were processed using fastp (V.0.19.7) to acquire clean data for subsequent analysis (29) and aligned to the *Mus musculus* genome assembly GRCm39 were removed using Bowtie (V2.4.5) to obtain metagenomic DNA sequences. Bowtie (V2.4.5) was used to align clean reads to EMGC database for quantification of corresponding gene abundances. These abundances were subsequently integrated to reconstruct the taxonomic composition profile of the metagenome (32). Whereas HUMAnN (V3.0.0. alpha.4) was used to estimate the functional pathways (33). Alpha-diversity analysis (Shannon index, Simpson index, Invsimpson index and Observed species) was conducted by vegan package of R (34), and the difference in alpha diversity between two groups were estimated with wilcoxon rank-sum test. Cecal microbiota and functional differences were conducted by LEfSe (LDA > 2, *p* < 0.05). Spearman’s correlation was used to describe the specific correlation between taxonomic groups. Raw data is available in NCBI Bioproject (Accession: PRJNA1333627).

### Statistical analysis

Statistical analysis of animal data was performed using GraphPad Prism (version 10.2.3), and data are presented as the mean ± standard deviation (SD) unless otherwise indicated. Sample sizes for animal experiments were not statistically predetermined but were similar to those reported in previously published works. Animals exhibiting abnormal behavior or physiological conditions were excluded before group assignment. Data from cell assays were processed and analyzed using Cytation5 software [version 3.11.19; (35)]. Normality was assessed using GraphPad Prism [version 10.2.3; (36)] before conducting comparisons. Outliers were identified and excluded using the ROUT method with Q = 1%. Investigators were not fully blinded during animal dosing and sample collection, and data collection and analysis were not performed blind to experimental conditions. Normality was tested using the Shapiro–Wilk test for n < 5 and the Kolmogorov–Smirnov test for *n* ≥ 5. Homogeneity of variance was assessed using the Brown–Forsythe test. For two-group comparisons, a two-tailed unpaired t-test was used when data were normally distributed and variances were equal; otherwise, the Mann–Whitney U test was applied. For multiple comparisons, one-way ANOVA followed by Dunnett’s *post hoc* test (with DIO_CK as control) was used for parametric data with equal variances. Welch’s ANOVA followed by Dunnett’s T3 test was used for parametric data with unequal variances. Non-parametric data were analyzed using the Kruskal–Wallis test followed by Dunn’s multiple comparison test. All graphical representations were generated using GraphPad Prism version 10.2.3.

## Results

### Viable MNO-863 improved obesity-related symptoms in HFD-induced obese mice

To find out *Christensenellaceae* species associated with metabolic disease therapies, firstly, we analyzed the frequency of occurrence (FO) and relative abundances (RA) of *Christensenellaceae* species in the downloaded published gut metagenomic datasets of metabolic disorders, such as obesity (OB), nonalcoholic fatty liver disease (NAFLD), type 2 diabetes (T2D) and Atherosclerotic cardiovascular disease (ACVD). We discovered that *Christensenellaceae* and nearly all *Christensenellaceae* species were prevalent across various study groups, and the abundance of *Christensenellaceae*, *Christensenella*, *C. minuta* and *C. intestinihominis* exhibited a significant decrease in all these 4 disease cohorts (Supplementary Figure S1). However, the cultured *Christensenellaceae* strain resources were limited, in our microbial bank, we obtained 208 *Christensenella* isolates from the fecal samples of healthy volunteers. Based on whole genome sequencing results, the comparison of COG (Cluster of Orthologous Groups) category average number between twelve *C. intestinihominis* strains and eighty-three *C. minuta* strains revealed these two species had similar function profile, while *C. intestinihominis* might have slightly stronger function of energy production and conversion, carbohydrate transport and metabolism, cell motility and inorganic ion transport and metabolism (Supplementary Figure S2). We selected four *Christensenella* strains including and other strains of *Akkermansia muciniphila* (77), *Akkermansia massiliensis* (17), *Bacteroides thetaiotaomicron* (23), *Parabacteroides distasoni* (37), *Intestinimonas butyricciproducens* (38), *Parabacteroides goldsteinii* (39), *Coprococcus comes* (40), *Blautia obeum* (41) and *Blautia wexlerae* (40) that have previously reported weight loss effects to intervene in obese mice and observe their weight loss effects, among which MNO-863 had the best weight loss effect (Supplementary Figure S3).

To evaluate if MNO-863 impacts obesity and metabolic disorders and whether its effects was dependent on the viable bacteria, we treated HFD-induced obese mice with vehicle buffer (HFD-Vehicle), Viable MNO-863 (MNO-863) or Heat-Killed MNO-863 (HK MNO-863) and treated with NCD mice with vehicle buffer as normal control, by oral gavage for a period of 4 weeks in experiment 1 (Figure 1A). MNO-863 significantly reduced body weight in obese mice (Figure 1B), as supported by 10.40% body weight reduction (Figure 1C). In contrast, no significant reduction in body weight was observed in HK MNO-863 (Figure 1B), which exhibited 0.68% body weight reduction (Figure 1C). Therefore, the effect of viable MNO-863 on body weight in diet-induced obese mice was significantly superior to that of Heat-Killed MNO-863. Besides, the mice in the MNO-863 group showed significantly reduced cumulative food intake (Figures 1D,E), fasting blood glucose and area under the curve (AUC) values in the oral glucose tolerance tests (OGTT, Figures 1F–H). The effects of MNO-863 on serum lipids for HFD-induced obese mice were further analyzed. Compared with HFD-Vehicle, MNO-863 significantly reduced TG, CHO and LDL (Table 1). By contrast, HK MNO-863 had no significant effect on serum lipids (Table 1). As shown in Table 1, MNO-863 significantly decreased liver weight, ALT and AST in obese mice. Whether viable MNO-863 was able to improve the severity of NASH lesions in the liver was further analyzed. MNO-863 significantly reduced liver steatosis, liver ballooning degeneration, and NAS score (Figures 1J–M), as supported by significant improved liver pathology and significantly reduced oil droplets (Figure 1I). In the MNO-863 group, there was a significant reduction in the weight of SAT (subcutaneous adipose tissue), EAT (epididymal adipose tissue), IAT (inguinal adipose tissue), and BAT (brown adipose tissues), reflecting its effect on body weight (Table 1). Since high-fat diet-induced obesity often leads to adipose tissue cell hypertrophy and lipid accumulation (24), we analyzed the effects of MNO-863 on white and brown adipose tissues (Figures 1N–R). MNO-863-treated mice showed smaller and fewer large adipocytes in epididymal and brown fat depots, with reduced average cell size. In contrast, heat-killed MNO-863 did not significantly alter cell size or pathology. Therefore, the data demonstrated that viable MNO-863 efficiently alleviates obesity and related metabolic disorders in diet-induced obesity and its effect is superior to that of the heat-killed MNO-863.

**Table 1:** Summary data of tissue weight and clinical chemistry in Experiment 1.

Indicators	Group			
	NCD-Vehicle	HFD-Vehicle	MNO-863	HK MNO-863
Liver weight (g)	0.89 ± 0.15***	1.13 ± 0.25	0.90 ± 0.09*	0.97 ± 0.10
SAT weight (g)	0.61 ± 0.17****	4.10 ± 0.61	3.11 ± 0.63*	3.62 ± 0.91
IAT weight (g)	0.13 ± 0.05****	1.22 ± 0.14	0.88 ± 0.23***	1.07 ± 0.20
EAT weight (g)	0.50 ± 0.09****	2.84 ± 0.26	2.60 ± 0.28	2.97 ± 0.18
BAT weight (g)	0.11 ± 0.01****	0.23 ± 0.04	0.17 ± 0.04**	0.21 ± 0.04
TG (mmol/L)	0.812 ± 0.116**	1.021 ± 0.114	0.821 ± 0.099*	1.076 ± 0.132
CHO (mmol/L)	3.306 ± 0.323****	6.564 ± 0.612	5.742 ± 0.532**	5.979 ± 1.015
LDL (mmol/L)	0.640 ± 0.036	0.659 ± 0.090	0.501 ± 0.106**	0.602 ± 0.098
HDL (mmol/L)	1.878 ± 0.253****	2.452 ± 0.142	2.545 ± 0.103	2.449 ± 0.350
ALT (U/L)	21.553 ± 2.186****	72.262 ± 38.416	25.676 ± 4.208****	35.244 ± 7.618
AST (U/L)	156.000 ± 43.191	187.360 ± 38.527	127.534 ± 8.674**	151.510 ± 35.133

Data are expressed as Mean ± SD. Statistical analysis using GraphPad software (version 10.2.3) was performed by one-way ANOVA combined with Dunnett’s multiple comparisons test. ns: not significant, not showed; *p < 0.05, **p < 0.01, ***p < 0.001 and ****p < 0.0001.

### MNO-863 improved obesity-related symptoms in HFD-induced obese mice in the dose-dependent manner

To explore the dose–response relationship of viable MNO-863 in the treatment of obesity and metabolic disorders, we treated HFD-induced obese mice with vehicle buffer (HFD-Vehicle), High dose (High-MNO-863, 1.2 × 10^11^ CFU/day), Middle dose (Middle-MNO-863, 2.4 × 10^10^ CFU/day), Low dose (Low-MNO-863, 2.4 × 10^9^ CFU/day) by oral gavage for a period of 28 days in experiment 2 (Figure 2A). High-MNO-863 and Middle-MNO-863 significantly reduced body weight in obese mice, as supported by 16.92 and 7.30% body weight reduction compared with HFD-Vehicle (Figures 2B,C). In contrast, no significant reduction in body weight was observed in Low-MNO-863, which exhibited 2.45% body weight reduction compared with HFD-Vehicle (Figures 2B,C). As shown in Figures 2D,E, High-MNO-863 significantly reduced food intake compared to HFD-Vehicle, with no change in Middle and Low-MNO-863 groups. High and Middle-MNO-863 significantly decreased OGTT AUC and High-MNO-863 also lowering fasting blood glucose (Figures 2F–H). High-MNO-863 reduced CHO and LDL, with no effects in Middle and Low-MNO-863 groups (Table 2). Further, High-MNO-863 notably reduced liver weight and ALT, while Middle-MNO-863 lowered ALT, with no effect in Low-MNO-863 (Table 2). Thus, MNO-863 showed a dose-dependent effect on weight management, glucose tolerance, lipid profile and liver function regulation in HFD-induced obese mice. Next, we studied how different MNO-863 doses affect lipid accumulation in liver (Figures 2I–M). High-MNO-863 also decreased liver steatosis, ballooning degeneration, and NAS scores compared to HFD-Vehicle, and Middle-MNO-863 reduced ballooning degeneration and NAS scores (Figures 2J–M), as supported by significant improved liver pathology and significantly reduced oil droplets (Figure 2I). Regarding adipose tissue, High-MNO-863 significantly lowered SAT, IAT, and BAT weights more than HFD-Vehicle, and Middle-MNO-863 significantly reduced SAT weight (Table 2). Our results indicated High-MNO-863 reduced EAT and BAT cell size, while Middle-MNO-863 reduced BAT cell size and High-MNO-863 increased small adipocytes and decreased large ones in epididymal and brown fat depots (Figures 2N–R). Thus, MNO-863 exhibited a dose-dependent effect, with the best results at 1.2 × 10^11^ CFU/day.

**Table 2:** Summary data of tissue weight and clinical chemistry in Experiment 2.

Indicators	Group			
	HFD-Vehicle	High-NMO-863	Middle-MNO-863	Low-MNO-863
Liver weight (g)	1.46 ± 0.34	1.06 ± 0.15***	1.25 ± 0.35	1.50 ± 0.34
SAT weight (g)	4.69 ± 1.06	2.98 ± 1.02***	3.35 ± 1.60**	4.93 ± 1.08
IAT weight (g)	1.22 ± 0.23	0.97 ± 0.29*	0.98 ± 0.33	1.01 ± 0.26
EAT weight (g)	2.43 ± 0.57	2.11 ± 0.59	2.20 ± 0.63	2.54 ± 0.65
BAT weight (g)	0.29 ± 0.05	0.19 ± 0.05****	0.24 ± 0.10	0.28 ± 0.06
TG (mmol/L)	0.841 ± 0.124	0.722 ± 0.113	0.900 ± 0.232	0.790 ± 0.148
CHO (mmol/L)	6.964 ± 0.547	5.884 ± 0.670**	6.291 ± 1.296	6.920 ± 0.672
LDL (mmol/L)	0.366 ± 0.095	0.292 ± 0.045*	0.370 ± 0.082	0.380 ± 0.055
HDL (mmol/L)	2.723 ± 0.236	2.778 ± 0.187	2.797 ± 0.343	2.894 ± 0.185
ALT (U/L)	131.458 ± 42.712	56.686 ± 29.070***	80.167 ± 53.356*	120.447 ± 75.383
AST (U/L)	199.036 ± 43.657	162.633 ± 32.047	163.681 ± 33.578	250.976 ± 83.827

Data are expressed as Mean±SD. Statistical analysis using GraphPad software (version 10.2.3) was performed by one-way ANOVA combined with Dunnett’s multiple comparisons test. ns: not significant, not showed; *p < 0.05, **p < 0.01, ***p < 0.001 and ****p < 0.0001.

Furthermore, the effects of viable MNO-863, Liraglutide, and their combination on weight management were investigated in HFD-induced obese mice in experiment 3 (Figure 3A). MNO-863, Liraglutide and MNO-863 + Liraglutide significantly decreased body weight, body weight gain, cumulative food intake, fasting blood glucose and AUC values in OGTT test, and their combination was more effective than either alone on weight management (Figures 3B–H). We also tested viable MNO-863 in another obesity model, administering it to HFHCD -induced obese mice in experiment 4 (Figure 3I). In HFHCD-induced mice, MNO-863, Liraglutide and MNO-863 + Liraglutide significantly decreased body weight, body weight gain, fasting blood glucose and AUC values in OGTT test, and their combination significantly decreased cumulative food intake (Figures 3J–P). The results confirmed that the combination was superior to MNO-863 or liraglutide alone in managing weight and improving hyperglycemia in obese mice on HFD or HFHCD diet (Figure 3).

### MNO-863 regulated serum biomarkers related to obesity in HFD-induced obese mice

**Figure 4:**
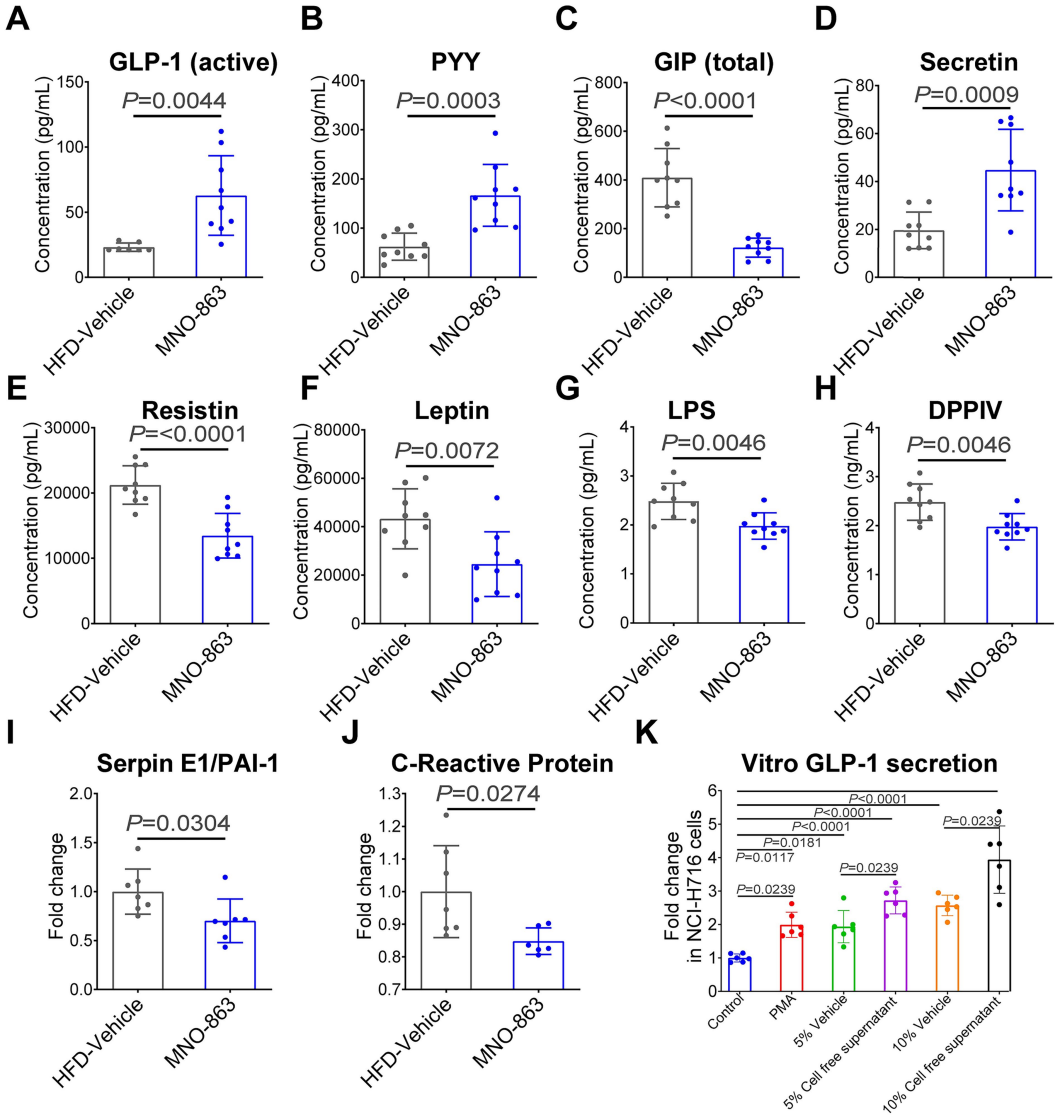
MNO-863 activated potential serum biomarkers in HFD-induced obese mice. **(A)** GLP-1 (active). **(B)** PYY. **(C)** GIP (total). **(D)** Secretin. **(E)** Resistin. **(F)** Leptin. **(G)** LPS. **(H)** DPPIV. **(I)** Serpin E1/PAI-1. **(J)** C-Reactive Protein. **(K)** GLP-1 secretion in NCI-H716 human intestinal L-cells (3 replicates per treatment and 2 replicates per detection). Data are presented as mean ± SD. Statistical analysis was performed using two-tail unpaired Student’s *t* test or one-way ANOVA combined with Dunnett’s multiple comparisons test **(K)**. ns: not significant, not showed; *p < 0.05, **p < 0.01, ***p < 0.001, ****p < 0.0001.

To explore the blood biomarkers of MNO-863 for weight loss and improvement of obesity-related complications, we further analyzed the level of factors related to appetite regulation, glucose and lipid metabolism and homeostasis, and inflammation in blood samples from experiment 1. As shown in Figure 4; Supplementary Table S1, compared with HFD-Vehicle, MNO-863 significantly increased GLP-1, PYY and secretin (Figures 4A–C), while MNO-863 significantly decreased GIP (Glucose-dependent Insulinotropic Polypeptide), resistin and leptin (Figures 4D–F). MNO-863 tended to increase amylin and ghrelin levels, and down-regulated C-peptide 2, insulin, and glucagon, however, no significant differences were observed (Supplementary Table S1). MNO-863 was analyzed for its effects on metabolic endotoxemia in obese mice and was found to significantly decrease plasma LPS level by 20.3% (Figure 4G and Supplementary Table S1). Further, MNO-863 significantly down-regulated the relative proportions of Dipeptidyl Peptidase IV (DPPIV, or CD26), Serpin E1/PAI-1 (Serpin family E member 1/Plasminogen activator inhibitor-1) and CRP (C-reactive protein) in serum by 34.1, 29.8, and 15.2%, respectively, but increased IGFBP-1 (Insulin-like growth factor-binding protein 1) up to 45.4% in comparison with HFD-Vehicle (Figures 4H–J and Supplementary Table S2).

To further confirm the mechanism of MNO-863 in the treatment of obesity and related complications, we evaluated the effect of MNO-863 on GLP-1 expression in NCI-H716 cells *in vitro* (Figure 4K). Compared to the 5% Vehicle group, MNO-863 5% CFS (cell-free supernatant) showed a trend towards increasing GLP-1 expression. Compared to the 10% Vehicle group, MNO-863 10% CFS significantly increased GLP-1 expression. MNO-863 CFS significantly induced the GLP-1 expression, suggesting that the induction of GLP-1 expression in intestinal cells by metabolites of the MNO-863 strain. Next, we will explore which molecules released by MNO-863 play a role in obesity and related complications management.

### MNO-863 regulated metabolites of cecal contents in HFD-induced obese mice

**Figure 5:**
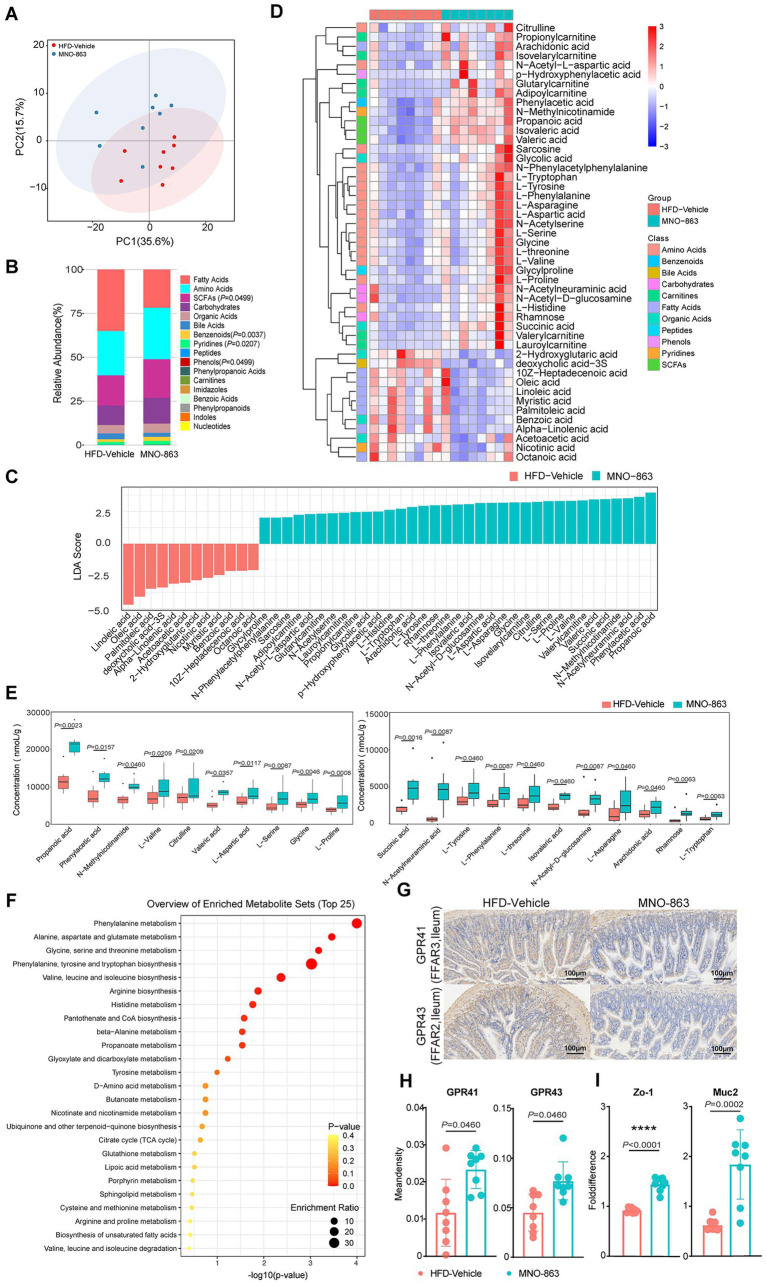
MNO-863 regulated metabolites of cecal contents in HFD-induced obese mice. **(A)** 2D score plot of OPLS-DA analysis of cecum content metabolites. **(B)** Relative abundance of each metabolite classes in different groups in cecum content metabolomics analysis. **(C)** LDA score of differential cecum content metabolites by lefse analysis. **(D)** Heatmap analysis of significantly differential cecum content metabolites. **(E)** Content of the main metabolites significantly up-regulated by MNO-863 (*n* = 8/group). **(F)** Bubble plots of MNO-863 enrichment pathway generated by MetaboAnalyst. **(G,H)** Representative immunohistochemistry images and quantitative analysis (Mean density) of GPR41 and GPR43 in ileum. **(I)** Relative expression of colonic ZO-1 and Muc-2. Data are presented as median **(E)** and mean ± SD **(H,I)** and Statistical analysis was performed using two-tail unpaired Student’s *t* test **(E,H,I)**. ns: not significant, not showed; *p < 0.05, **p<0.01, ***p < 0.001,****p < 0.0001.

To find out which metabolites are associated with GLP-1 production in gut, weight loss and improved metabolic health, the cecal metabolites in MNO-863-treated obese mice were investigated by performing a targeted metabolomic analysis. As shown in Figure 5A, the PCA score plots of HFD-Vehicle and MNO-863 showed intra-group aggregation and significant inter-group separation (PC2 Wilcoxon rank-sum test *p* value = 0.006993), indicating significant differences in metabolite composition between the two groups. As shown in Figure 5B, MNO-863 significantly up-regulated the abundance of SCFAs, benzenoids, pyridines, and phenols compounds among seventeen main metabolites classes (Supplementary File S1). The differential metabolites were further identified by unidimensional test based on *p* < 0.05, revealing 47 significantly differential metabolites (Figure 5C; Supplementary File S1). Compared with HFD-Vehicle, MNO-863 significantly up-regulated 35 differential metabolites and down-regulated 12 differential metabolites (Figure 5C; Supplementary File S1). Heatmap analysis was further performed with the Z-values after the normalization of data of metabolomic differential metabolites of cecum contents (Figure 5D; Supplementary File S1). By contrast, MNO-863 significantly down-regulated 12 differential metabolites, among which myristoleic acid (42), DCA (deoxycholic acid−3S) (43), myristic acid (42) and nicotinic acid (44) had been previously reported to be positively correlated with disease progression. MNO-863 up-regulated 35 differential metabolites including propionic acid (45), isovaleric acid (46), N-acetylneuraminic acid (47) and succinic acid (48), which was positively correlated with the improvement of metabolic diseases or demonstrated to reduce obesity and its related disorders. Among these increased metabolites, propionic acid and isovaleric acid stands out, with 84.9 and 58.1%, which benefits for the control of body weight gain and amelioration of the insulin sensitivity (Figure 5E; Supplementary File S1). Bubble plots generated by MetaboAnalyst unveiled MNO-863’s impact on those enriched metabolites and highlighted significantly altered pathways, including amino acid metabolism (e.g., phenylalanine metabolism) (Figure 5F; Supplementary File S1). Specifically, MNO-863 enrichment was observed in propionate-related pathways such as alanine/aspartate/glutamate metabolism, propanoate metabolism, and the citrate cycle (TCA cycle). Subsequent analysis focused on the abundance of key metabolites within these pathways. In addition, the expression of GPR41 and GPR43 was significantly increased in the MNO-863 group (Figures 5G,H). The expression of intestinal barrier genes ZO-1 and MUC2 were increased by treatment of MNO-863 (Figure 5I). The data above suggested that MNO-863 may exert the effect of weight management by increasing health-benefiting gut metabolites, especially SCFAs.

### MNO-863 remodeled gut microbiota and increased SCFAs-producing bacteria in HFD-induced obese mice

**Figure 6:**
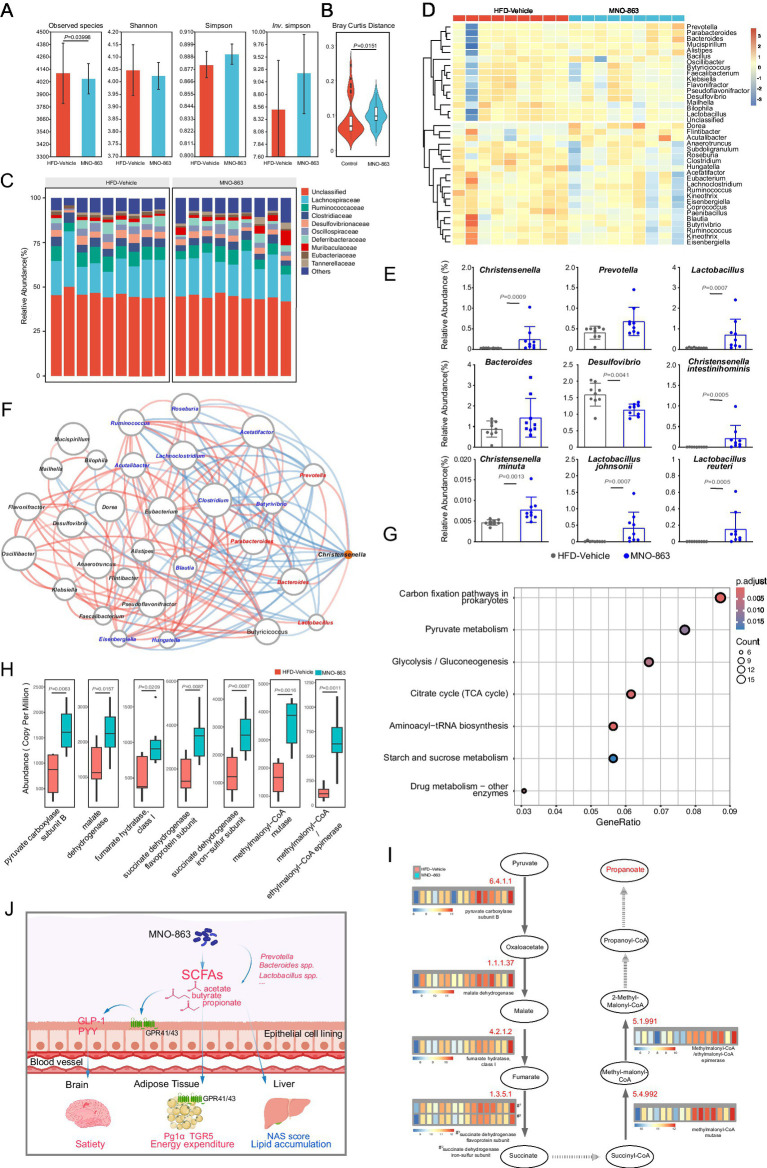
MNO-863 remodeled gut microbiota and increased SCFAs-producing bacteria in HFD-induced obese mice. **(A)** *α*-diversity. **(B)** PCOA at the genus level. **(C)** Relative abundances of taxonomic profiling at Family level. **(D)** Heatmap of the top35 genera by abundance. **(E)** Relative abundance of key SCFAs-producing bacteria or representative harmful bacteria regulated by MNO-863 administration. **(F)** Network diagram of the most abundant genera associated with Christensenella. **(G)** KEGG enrichment result of significant KEGG orthology. **(H)** Up-regulated KEGG orthology related to propionic acid synthesis (*n* = 8/group). **(I)** Putative propionic acid pathway regulated by MNO-863. **(J)** The mechanism model diagram of MNO-863 improves obesity-related symptoms. Data are presented as mean ± SD **(E)** and median **(H)**, and Statistical analysis was performed using two-tail unpaired Student’s *t* test **(E,H)**. ns: not significant, not showed; *p < 0.05, **p<0.01.

Given the significant changes of cecal metabolites by MNO-863, firstly, the content of short-chain fatty acids in MNO-863 fermentation broth was detected by HPLC and MNO-863 could metabolize to produce acetic acid and butyric acid (Supplementary Figure S4). Furthermore, the metagenome sequencing was performed to further explore the effects of MNO-863 on gut microbiota composition and function in HFD-induced obese mice of experiment 1. As shown in Figure 6A, no significant differences were observed between the two groups in the analysis of Shannon and Simpson and Inv Simpson diversity indicators, and the observed species decreased. By contrast, MNO-863 tended to increase richness and evenness with an increase in Simpson and *Inv.* Simpson. The *β*-diversity of cecal microbiota composition was further analyzed by Bray Curtis distance. Compared with HFD-Vehicle, MNO-863 significantly altered β-diversity at the genus level (Figure 6B). No significant alteration in relative abundances of taxonomic profiling at family level was observed between MNO-863 and HFD-Vehicle groups (Figure 6C). Oral administration of MNO-863 promotes the abundance of *Christensenella, Prevotella, Lactobacillus and Bacteroides* at genus level, and the abundance of probiotics *Limosilactobacillus johnsonii* and *Limosilactobacillus reuteri* at species level and reduced the abundance of endotoxin-producing *Proteobacteria* and *Desulfovibrio* (Figures 6D,E; Supplementary File S2). In early studies, the genera of *Christensenella* (17, 18), *Lactobacillus* (49), *Prevotella* (50), *Bacteroides* (24), *Butyricicoccus* (51) have been reported to be associated with improved obesity or related complications, and the species of *C. minuta* (17, 18, 21), *Limosilactobacillus johnsonii* (49), and *Limosilactobacillus reuteri* (52) have been confirmed to reduce obesity *in vivo* animal assays. In addition, species in the genus of *Christensenella*, *Prevotella*, *Lactobacillus* and *Bacteroides* have shown ability in generating SCFAs, with members of *Prevotella* specializing in production of propionic acid (50, 53). The genus and species significantly down-regulated by MNO-863 in the gut flora including *Robinsoniella* (54), *Romboutsia* (55), *Bilophila* (56), *Desulfovibrio* (57), *Romboutsia ilealis* (49), *Desulfovibrio piger* (57) and *Anaerotruncus colihominis* (53) were found to be associated with the progression of obesity-related diseases. A further networking analysis on the bacterial genus showed the positive correlations between *Christensenella* with *Lactobacillus*, *Prevotella*, *Parabacteroides* and *Bacteroides* (Figure 6F; Supplementary File S2). To characterize bacterial gene pathways potentially mediating weight loss and propionate accumulation, KEGG pathway enrichment analysis was performed. MNO-863 intervention significantly augmented three bacterial metabolic pathways, with citrate cycle (TCA cycle) and carbon fixation in prokaryotes showing the strongest association with elevated succinate and propionate levels (FDR < 0.05) (Figure 6G; Supplementary File S2). Integrative metagenomic-metabolomic analysis demonstrated that MNO-863 activated pyruvate metabolism and the tricarboxylic acid (TCA) cycle, channeling pyruvate into the key intermediate succinate. Subsequent activation of propanoate metabolism further converted succinate to propionate. Abundance profiling of pathway-specific KEGG Orthologs (KOs) via heatmap and boxplot visualization revealed significant enrichment of propanoate metabolism-related genes in the MNO-863 group, mechanistically linking microbiota reprogramming to enhanced propionate production (Figures 6H,I; Supplementary File S2). The above data suggested that MNO-863 may orchestrate the protective gut microbiota composition in HFD-induced obese mice via significantly increasing beneficial bacteria associated with improvement in obesity or related complications, such as increasing the abundance of health-benefiting bacteria, and decreasing harmful bacteria associated with obesity-related disease progression and endotoxin production.

### No adverse effects of MNO-863 in a repeated dose toxicity study in Sprague Dawley rats

To evaluate the potential toxicity and toxicokinetic characteristics of MNO-863 Fermental Powder when administered to SD rats twice daily for 28 consecutive days via oral gavage, and to observe the reversibility of these adverse effects and possible delayed toxicity following a 28-day recovery period, the toxicity study was conducted as follows: SD rats were orally administered with MNO-863 Fermental Powder at doses of 1.2 × 10^11^, 6 × 10^11^ and 1.2 × 10^12^ CFU/animal/day, respectively, and then allowed to recover for 28 days. All animals survived to the scheduled day of necropsy and there were no unplanned dead or moribund animals in this study. Compared with the results of the negative control group in the same period, male and female animals in each test article group showed no significant difference of body weight and food consumption during the dosing period and during the recovery period (Supplementary Figure S5 and Supplementary File S3).

Summary data of organ weight and clinical pathology analysis is presented in Supplementary Tables S3, S4 and Supplementary File S3. As a result, administration of MNO-863 produced no observed adverse effects on host. In the toxicokinetic study, the target bacteria MNO-863 of the live bacteria drugs of test article were not absorbed into the blood. Under the condition of this study, the no observed adverse effect level (NOAEL) of MNO-863 Fermental Powder was equal to or greater than 1.2 × 10^12^ CFU/animal/day. By considering this NOAEL and by applying an uncertainty factor of 200 (10 (interspecies variability) × 10 (intraspecies variability) × 2 (subchronic to chronic study duration)) and the average weight of the rats is 330 g. A safe level was equal to or greater than 1.5 × 10^10^ CFU/kg/day. For the target population (adults excluding pregnant and lactating women) with a default body weight of 70 kg (EFSA Scientific Committee, 2012), the safety margin was equal to or greater than 1.0 × 10^12^ CFU/day.

## Discussion

Studies have highlighted a clear association between the gut microbiome and metabolic disorders like obesity, diabetes, fatty liver disease, and cardiovascular diseases (58). The family of *Christensenellaceae* has been revealed to be negatively associated with obesity indicators such as body fat distribution, waist circumference, blood lipid levels, and blood pressure (17, 18). Goodrich et al. (18) investigated the gut microbiota of British twins and found that *Christensenellaceae* are more abundant in individuals with a low body mass index (BMI). This finding was corroborated by metagenomic studies of fecal samples from Japanese, Mexican, Spanish, and Korean populations (17). Among the family of *Christensenellaceae*, strains of *C. minuta* that was firstly isolated from healthy human feces were well investigated for its anti-obesity effects and the underlying mechanisms (20). In a study, it was observed that mice receiving the *C. minuta*-supplemented fecal transplants gained less weight and had lower body fat compared to those receiving only the obese subjects’ fecal microbiota (18). In another work, administration of the viable *C. minuta* reduced body weight gain, hyperglycemia, hyperlipidemia, lipids, and liver fat accumulation (20). YSOPIA Bioscience, a French company specializing in microbiome-based drugs, has developed Xla1, an experimental drug using *C. minuta*, to treat obesity and metabolic syndrome, which has completed Phase I clinical trial (NCT04663139) and demonstrated good safety and efficacy in reducing LDL cholesterol levels in the trial’s preliminary unpublished results (20). In this study, we analyzed *Christensenellaceae*’s occurrence and abundance in gut metagenomic datasets from obesity, NAFLD, T2D and ACVD patients. It was found *Christensenellaceae* species, were less abundant in these diseases compared to healthy individuals. Furthermore, a library including 208 *Christensenella* strains was established. *C. intestinihominis* MNO-863 was selected for further *in vivo* evaluation based on results of the genome analysis and preliminary screening. In further multi-model pharmacodynamic evaluation, MNO-863 has been demonstrated to be effective in the treatment of obesity and the obesity-related metabolic dysfunctions by significantly reducing body weight and improving glucose tolerance and lipid metabolic disorders in obese mice induced by high-fat diet. A dose–response relationship trend was also revealed for the efficacy of MNO-863.

The mechanisms by which gut commensal microbes improve metabolic diseases include lowering the systematic inflammatory responses, enhancing the energy expenditure, regulating metabolic signaling pathways, correcting the dysbiosis of gut microbiota (59). Gut microbial metabolic products such as short-chain fatty acids and secondary bile acids can activate or regulate host metabolic signaling pathways to reduce obesity and improve metabolic disorders (10). In one recent study, *C. minuta* has been confirmed to produce a novel class of secondary bile acid, 3-O-acylcholic acids, which targets and inhibits the farnesoid X receptor (FXR) in the gut, thereby modulating the enterohepatic axis and significantly ameliorating metabolic disorders in model mice (21). The genomic analysis of MNO-863 revealed that, unlike *C. minuta*, it lacks the bile acid-related genes that have been identified, such as bile salt hydrolase (BSH) gene. In the current work, oral administration of MNO-863 effectively elevated the levels of GLP-1, PYY, secretin and IGFBP-1, while decreased GIP, resistin, leptin, DPPIV, Serpin E1/PAI-1 and CRP. GLP-1, PYY and secretin belong to the hormones with pleiotropic effects, such as slowing gastric emptying, increasing satiety, increasing energy expenditure and thermogenesis as well as homeostatic effects on glucose and lipid metabolisms (14, 15). By contrast, down-regulation of GIP and resistin and leptin had been reported to be beneficial for obesity or diabetes treatment (14, 15). *In vitro* assay, the culture supernatant of MNO-863 notably induced GLP-1 expression in NCI-H716 cells, indicating MNO-863 may enhance the intestinal GLP-1 production, which together with in vivo data suggest the regulation of intestinal hormones as action mechanism for the anti-obesity efficacy of MNO-863.

The multi-omics integration analysis of gut microbiome and metabolome have been successfully applied in the elucidation of gut microbiota-dependent mechanisms for probiotics and prebiotics. For instance, the gut bacterium *Parabacteroides merdae*, protects against cardiovascular damage by enhancing the catabolism of branched-chain amino acids (BCAAs) (60). *Parabacteroides distasonis* alleviates obesity and metabolic disorders in mice by producing succinate and secondary bile acids, which activate intestinal gluconeogenesis and the FXR pathway (37). In the study of prebiotics, polysaccharides from *Lyophyllum decastes* reduce obesity in mice by altering the gut microbiota, enriching beneficial bacteria like *Bacteroides intestinalis* and *Lactobacillus johnsonii*, and enhancing energy expenditure via the bile acid-TGR5 pathway (61). Herein, a targeted metabolomic analysis revealed that MNO-863 up-regulated 35 metabolites potentially benefiting weight loss and diabetes. MNO-863 significantly increased the levels of SCFAs, especially the abundance of gut propionic acid, and butyric acid. Early studies have demonstrated that propionic acid and butyric acid can modulate the secretion of appetite- regulating hormones (such as GLP-1 and PYY) and insulin through activation of GPCRs. Additionally, SCFAs can enhance gut barrier function by inhibiting histone deacetylases (HDACs), promote fatty acid oxidation via the activation of peroxisome proliferator - activated receptor - *γ* (PPARγ) (62). MNO-863 shows capability of producing acetic acid and butyric acid *in vitro* fermentation. Consistently, cecal acetate increased from 59,971.13 ± 25,527.57 nmol/g in controls to 77,203.11 ± 21,551.25 nmol/g in the MNO-863 group, implying a contributory role. We now explicitly recognize that acetate may synergize with propionate and butyrate by activating AMPK, accelerating fat oxidation, refining glucose homeostasis, stimulating anorexigenic GLP-1 and PYY release, and engaging central satiety pathways (63, 64), thereby amplifying the overall metabolic benefits conferred by MNO-863.

Additionally, analysis of the gut microbiome revealed that MNO-863 elevated the abundance of *Prevotella*, *Lactobacillus*, and *Bacteroides*. Studies have demonstrated that *Prevotella* can selectively break down long-chain isomaltooligosaccharides (IMOs), resulting in increased SCFAs concentrations. Specifically, after IMO fermentation, the *Prevotella* -type microbiome shows greater production of propionic and butyric acids than the non-Prevotella -type microbiome (65). Another study revealed that *Prevotella ruminicola, Clostridium propionicum,* and *Megasphaera elsdenii* can utilize the acrylate pathway to produce propionate through the ATP-neutral conversion of lactate to propionate (66). Moreover, numerous studies have underscored the crucial role of Lactobacillus in SCFAs production, as well as its potential for weight management and regulation of blood glucose and lipids, exemplified by *L. reuter* (67), *L. plantarum* (68), *L. rhamnosus* (69) and so on. Obese mice exhibit a substantial decrease in *Bacteroides* species, which helps protect against excessive fat accumulation. Introducing *Bacteroides thetaiotaomicron* to mice on a normal diet significantly decreased their total fat content and prevented weight gain in those on a high-fat diet (70). *Bacteroides stercoris* KGMB02265 has been shown to inhibit lipid accumulation in 3 T3-L1 preadipocytes and reduce body and fat weight while improving glucose sensitivity in high-fat diet-induced obese mice (71). Furthermore, combining *Bacteroides uniformis* CECT 7771 with wheat bran extract enhances anti-obesity effects in diet-induced obese mice. A 17-week study demonstrated that this combination was particularly effective in curbing weight gain and adiposity, improving glucose disposal, restoring insulin-dependent metabolic pathways, and boosting butyrate production and intestinal immune defense (72). In line with the gut microbiome changes, functional genes involved in the biosynthesis of propionic acid within succinate pathway were enriched by MNO-863. Although our current study demonstrated that *C. intestinihominis* MNO-863—originally isolated from a healthy human donor and capable of transient colonization in the murine gut—can ameliorate obesity-associated metabolic disorders, several limitations must be acknowledged. These include the short treatment duration, the absence of causal evidence for the proposed mechanisms, and the inherent constraints of rodent models whose microbiomes differ markedly from that of humans (73, 74). Moreover, human gut microbiota composition varies substantially with ethnicity and geography, further complicating extrapolation of our animal findings (74, 75). Consequently, rigorous longer-term studies and well-controlled clinical trials are essential before MNO-863 can be considered for species-specific, bacterium-targeted management of metabolic diseases.

In conclusion, *C. intestinihominis* MNO-863 produced acetic acid and butyric acid itself and modulated gut microbiota to increase SCFAs-producing bacteria of *Prevotella*, *Lactobacillus*, and *Bacteroides*, which together increased the levels of butyrate acid and propionic acids. The elevated gut SCFAs, especially propionic acid, were accompanied by up-regulation of GPR41 and GPR43 and a parallel rise in the obesity-related hormones GLP-1 and PYY; a direct causal chain remains to be proven, mitigating the obesity and its associated metabolic disorders (Figure 6J). MNO-863 has been approved by FDA (Food and Drug Administration) and CDE (Center for Drug Evaluation) for clinical trials as a new Class I drug and is expected to provide a new, safer drug option for patients with obesity and related complications.

## Data Availability

The datasets presented in this study can be found in online repositories. The patient metagenomic sequencing data is previously published and publicly available in the NCBI repository under accession numbers: PRJEB12123 (https://www.ncbi.nlm.nih.gov/bioproject/324059), PRJEB21528 (https://www.ncbi.nlm.nih.gov/bioproject/324059), PRJNA422434 (https://www.ncbi.nlm.nih.gov/bioproject/422434), PRJNA373901 (https://www.ncbi.nlm.nih.gov/bioproject/373901), PRJNA278393 (https://www.ncbi.nlm.nih.gov/bioproject/278393) and PRJNA388263 (https://www.ncbi.nlm.nih.gov/bioproject/388263). Animal metabolomics data is publicly available in MetaboLights under accession number MTBLS13079 (https://www.ebi.ac.uk/metabolights/editor/MTBLS13079/descriptors). Raw sequencing data of microbiome is available in the NCBI GenBank repository under accession number PRJNA1333627 (https://www.ncbi.nlm.nih.gov/bioproject/1333627) and the sequencing data of the bacterial 16S rRNA genes is available in NCBI GenBank under accession number: PX470668.

## References

[1] Piche ME Tchernof A Despres JP. Obesity phenotypes, diabetes, and cardiovascular diseases. Circ Res. (2020) 126:1477–500. doi: 10.1161/CIRCRESAHA.120.316101, PMID: 32437302

[2] Wu Q Pi X Liu W Chen H Yin Y Yu HD . Fermentation properties of isomaltooligosaccharides are affected by human fecal enterotypes. Anaerobe. (2017) 48:206–14. doi: 10.1016/j.anaerobe.2017.08.016, PMID: 28882708

[3] Hall KD Kahan S. Maintenance of lost weight and long-term Management of Obesity. Med Clin North Am. (2018) 102:183–97. doi: 10.1016/j.mcna.2017.08.012, PMID: 29156185 PMC5764193

[4] Khera R Murad MH Chandar AK Dulai PS Wang Z Prokop LJ . Association of pharmacological treatments for obesity with weight loss and adverse events: a systematic review and meta-analysis. JAMA. (2016) 315:2424–34. doi: 10.1001/jama.2016.7602, PMID: 27299618 PMC5617638

[5] Arterburn DE Telem DA Kushner RF Courcoulas AP. Benefits and risks of bariatric surgery in adults: a review. JAMA. (2020) 324:879–87. doi: 10.1001/jama.2020.12567, PMID: 32870301

[6] Fackelmann G Manghi P Carlino N Heidrich V Piccinno G Ricci L . Gut microbiome signatures of vegan, vegetarian and omnivore diets and associated health outcomes across 21,561 individuals. Nat Microbiol. (2025) 10:41–52. doi: 10.1038/s41564-024-01870-z, PMID: 39762435 PMC11726441

[7] Yin Q da Silva AC Zorrilla F Almeida AS Patil KR Almeida A. Ecological dynamics of Enterobacteriaceae in the human gut microbiome across global populations. Nat Microbiol. (2025) 10:541–53. doi: 10.1038/s41564-024-01912-6, PMID: 39794474 PMC11790488

[8] Pedersen HK Gudmundsdottir V Nielsen HB Hyotylainen T Nielsen T Jensen BA . Human gut microbes impact host serum metabolome and insulin sensitivity. Nature. (2016) 535:376–81. doi: 10.1038/nature18646, PMID: 27409811

[9] Zheng L Ji YY Wen XL Duan SL. Fecal microbiota transplantation in the metabolic diseases: current status and perspectives. World J Gastroenterol. (2022) 28:2546–60. doi: 10.3748/wjg.v28.i23.2546, PMID: 35949351 PMC9254144

[10] Zhao L. The gut microbiota and obesity: from correlation to causality. Nat Rev Microbiol. (2013) 11:639–47. doi: 10.1038/nrmicro3089, PMID: 23912213

[11] De Vadder F Kovatcheva-Datchary P Goncalves D Vinera J Zitoun C Duchampt A . Microbiota-generated metabolites promote metabolic benefits via gut-brain neural circuits. Cell. (2014) 156:84–96. doi: 10.1016/j.cell.2013.12.016, PMID: 24412651

[12] Wahlstrom A Sayin SI Marschall HU Backhed F. Intestinal crosstalk between bile acids and microbiota and its impact on host metabolism. Cell Metab. (2016) 24:41–50. doi: 10.1016/j.cmet.2016.05.005, PMID: 27320064

[13] Dodd D Spitzer MH Van Treuren W Merrill BD Hryckowian AJ Higginbottom SK . A gut bacterial pathway metabolizes aromatic amino acids into nine circulating metabolites. Nature. (2017) 551:648–52. doi: 10.1038/nature24661, PMID: 29168502 PMC5850949

[14] Tolhurst G Heffron H Lam YS Parker HE Habib AM Diakogiannaki E . Short-chain fatty acids stimulate glucagon-like peptide-1 secretion via the G-protein-coupled receptor FFAR2. Diabetes. (2012) 61:364–71. doi: 10.2337/db11-1019, PMID: 22190648 PMC3266401

[15] Karra E Batterham RL. The role of gut hormones in the regulation of body weight and energy homeostasis. Mol Cell Endocrinol. (2010) 316:120–8. doi: 10.1016/j.mce.2009.06.010, PMID: 19563862

[16] Chen Y Zhang J Li P Liu C Li L. N1-methylnicotinamide ameliorates insulin resistance in skeletal muscle of type 2 diabetic mice by activating the SIRT1/PGC-1alpha signaling pathway. Mol Med Rep. (2021) 23:270. doi: 10.3892/mmr.2021.11909 33576435

[17] Waters JL Ley RE. The human gut bacteria Christensenellaceae are widespread, heritable, and associated with health. BMC Biol. (2019) 17:83. doi: 10.1186/s12915-019-0699-4, PMID: 31660948 PMC6819567

[18] Goodrich JK Waters JL Poole AC Sutter JL Koren O Blekhman R . Human genetics shape the gut microbiome. Cell. (2014) 159:789–99. doi: 10.1016/j.cell.2014.09.053, PMID: 25417156 PMC4255478

[19] Zou Y Xue W Lin X Hu T Liu SW Sun CH . Taxonomic description and genome sequence of *Christensenella intestinihominis* sp. nov., a novel cholesterol-lowering bacterium isolated from human gut. Front Microbiol. (2021) 12:632361. doi: 10.3389/fmicb.2021.632361, PMID: 33692769 PMC7937921

[20] Mazier W Le Corf K Martinez C Tudela H Kissi D Kropp C . A new strain of *Christensenella minuta* as a potential biotherapy for obesity and associated metabolic diseases. Cells. (2021) 10:823. doi: 10.3390/cells10040823, PMID: 33917566 PMC8067450

[21] Liu L Chen Y Wu Q Shu A Sun J. Sodium butyrate attenuated diabetes-induced intestinal inflammation by modulating gut microbiota. Evid Based Complement Alternat Med. (2022) 2022:1–12. doi: 10.1155/2022/4646245, PMID: 36045662 PMC9423962

[22] Wang Y Luo B Wu XQ Li X Liao SJ. Comparison of the effects of tai chi and general aerobic exercise on weight, blood pressure and glycemic control among older persons with depressive symptoms: a randomized trial. BMC Geriatr. (2022) 22:401. doi: 10.1186/s12877-022-03084-6, PMID: 35525971 PMC9077840

[23] Gao X Xie Q Kong P Liu L Sun S Xiong B . Polyphenol- and caffeine-rich Postfermented Pu-erh tea improves diet-induced metabolic syndrome by remodeling intestinal homeostasis in mice. Infect Immun. (2018) 86:e00601-17. doi: 10.1128/IAI.00601-17, PMID: 29061705 PMC5736808

[24] Kleiner DE Brunt EM Van Natta M Behling C Contos MJ Cummings OW . Design and validation of a histological scoring system for nonalcoholic fatty liver disease. Hepatology. (2005) 41:1313–21. doi: 10.1002/hep.20701, PMID: 15915461

[25] Jang H Lim H Park KH Park S Lee HJ. Changes in plasma choline and the betaine-to-choline ratio in response to 6-month lifestyle intervention are associated with the changes of lipid profiles and intestinal microbiota: the ICAAN study. Nutrients. (2021) 13:4006. doi: 10.3390/nu13114006, PMID: 34836260 PMC8625635

[26] Dyck S Kataria H Alizadeh A Kallivalappil TS Lang B Silver J . Perturbing chondroitin sulfate proteoglycan signaling through LAR and PTPσ receptors promotes a beneficial inflammatory response following spinal cord injury. J Neuroinflammation. (2018) 15:90. doi: 10.1186/s12974-018-1128-2 29558941 PMC5861616

[27] Ma XJ Gao YX Ren ZB Dong H Zhang X Niu NK. Study on the role and molecular mechanism of METTL3-mediated miR-29a-3p in the inflammatory response of spinal tuberculosis. Tuberculosis (Edinb). (2024) 148:102546. doi: 10.1016/j.tube.2024.102546, PMID: 39079219

[28] Yoon HS Cho CH Yun MS Jang SJ You HJ Kim JH . *Akkermansia muciniphila* secretes a glucagon-like peptide-1-inducing protein that improves glucose homeostasis and ameliorates metabolic disease in mice. Nat Microbiol. (2021) 6:563–73. doi: 10.1038/s41564-021-00880-5, PMID: 33820962

[29] Chen S Zhou Y Chen Y Gu J. fastp: an ultra-fast all-in-one FASTQ preprocessor. Bioinformatics. (2018) 34:i884–90. doi: 10.1093/bioinformatics/bty560, PMID: 30423086 PMC6129281

[30] Erawijantari PP Mizutani S Shiroma H Shiba S Nakajima T Sakamoto T . Influence of gastrectomy for gastric cancer treatment on faecal microbiome and metabolome profiles. Gut. (2020) 69:1404–15. doi: 10.1136/gutjnl-2019-319188, PMID: 31953253 PMC7398469

[31] Tringe SG von Mering C Kobayashi A Salamov AA Chen K Chang HW . Comparative metagenomics of microbial communities. Science. (2005) 308:554–7. doi: 10.1126/science.1107851, PMID: 15845853

[32] Feng Q Liang S Jia H Stadlmayr A Tang L Lan Z . Gut microbiome development along the colorectal adenoma–carcinoma sequence. Nat Commun. (2015) 6:6528. doi: 10.1038/ncomms7528, PMID: 25758642

[33] Franzosa EA McIver LJ Rahnavard G Thompson LR Schirmer M Weingart G . Species-level functional profiling of metagenomes and metatranscriptomes. Nat Methods. (2018) 15:962–8. doi: 10.1038/s41592-018-0176-y, PMID: 30377376 PMC6235447

[34] Oksanen J Simpson G Blanchet F . (2019) vegan: community ecology package. R package, 2019. Available online at: https://CRAN.R-project.org/package=vegan

[35] Hubbard WB Velmurugan GV Brown EP Sullivan PG. Resilience of females to acute blood-brain barrier damage and anxiety behavior following mild blast traumatic brain injury. Acta Neuropathol Commun. (2022) 10:93. doi: 10.1186/s40478-022-01395-8, PMID: 35761393 PMC9235199

[36] Xian Y Chen Z Lan Z Zhang C Sun H Liu Z . Live biotherapeutic *enterococcus lactis* MNC-168 promotes the efficacy of immune checkpoint blockade in cancer therapy by activating STING pathway via bacterial membrane vesicles. Gut Microbes. (2025) 17:2557978. doi: 10.1080/19490976.2025.2557978, PMID: 40937720 PMC12439585

[37] Wang K Liao M Zhou N Bao L Ma K Zheng Z . *Parabacteroides distasonis* alleviates obesity and metabolic dysfunctions via production of succinate and secondary bile acids. Cell Rep. (2019) 26:222–235.e5. doi: 10.1016/j.celrep.2018.12.028, PMID: 30605678

[38] Rampanelli E Romp N Troise AD Ananthasabesan J Wu H Gul IS . Gut bacterium Intestinimonas butyriciproducens improves host metabolic health: evidence from cohort and animal intervention studies. Microbiome. (2025) 13:15. doi: 10.1186/s40168-024-02002-9, PMID: 39833973 PMC11744835

[39] Wu TR Lin CS Chang CJ Lin TL Martel J Ko YF . Gut commensal *Parabacteroides goldsteinii* plays a predominant role in the anti-obesity effects of polysaccharides isolated from *Hirsutella sinensis*. Gut. (2019) 68:248–62. doi: 10.1136/gutjnl-2017-315458, PMID: 30007918

[40] Guo Y Huang ZP Liu CQ Qi L Sheng Y Zou DJ. Modulation of the gut microbiome: a systematic review of the effect of bariatric surgery. Eur J Endocrinol. (2018) 178:43–56. doi: 10.1530/EJE-17-0403, PMID: 28916564

[41] Wang H Lv X Zhao S Yuan W Zhou Q Sadiq FA . Weight loss promotion in individuals with obesity through gut microbiota alterations with a multiphase modified ketogenic diet. Nutrients. (2023) 15:4163. doi: 10.3390/nu15194163, PMID: 37836447 PMC10574165

[42] Saraswathi V Kumar N Ai W Gopal T Bhatt S Harris EN . Myristic acid supplementation aggravates high fat diet-induced adipose inflammation and systemic insulin resistance in mice. Biomolecules. (2022) 12:739. doi: 10.3390/biom12060739, PMID: 35740864 PMC9220168

[43] Sato Y Atarashi K Plichta DR Arai Y Sasajima S Kearney SM . Novel bile acid biosynthetic pathways are enriched in the microbiome of centenarians. Nature. (2021) 599:458–64. doi: 10.1038/s41586-021-03832-5, PMID: 34325466

[44] Kroon T Baccega T Olsen A Gabrielsson J Oakes ND. Nicotinic acid timed to feeding reverses tissue lipid accumulation and improves glucose control in obese Zucker rats[S]. J Lipid Res. (2017) 58:31–41. doi: 10.1194/jlr.M068395, PMID: 27875257 PMC5234709

[45] Heimann E Nyman M Degerman E. Propionic acid and butyric acid inhibit lipolysis and de novo lipogenesis and increase insulin-stimulated glucose uptake in primary rat adipocytes. Adipocyte. (2015) 4:81–8. doi: 10.4161/21623945.2014.960694, PMID: 26167409 PMC4496978

[46] Heimann E Nyman M Palbrink AK Lindkvist-Petersson K Degerman E. Branched short-chain fatty acids modulate glucose and lipid metabolism in primary adipocytes. Adipocyte. (2016) 5:359–68. doi: 10.1080/21623945.2016.1252011, PMID: 27994949 PMC5160390

[47] Yida Z Imam MU Ismail M Ismail N Azmi NH Wong W . N-Acetylneuraminic acid supplementation prevents high fat diet-induced insulin resistance in rats through transcriptional and nontranscriptional mechanisms. Biomed Res Int. (2015) 2015:602313. doi: 10.1155/2015/602313, PMID: 26688813 PMC4673348

[48] De Vadder F Kovatcheva-Datchary P Zitoun C Duchampt A Backhed F Mithieux G. Microbiota-produced succinate improves glucose homeostasis via intestinal gluconeogenesis. Cell Metab. (2016) 24:151–7. doi: 10.1016/j.cmet.2016.06.013, PMID: 27411015

[49] Rodrigues RR Gurung M Li Z Garcia-Jaramillo M Greer R Gaulke C . Transkingdom interactions between lactobacilli and hepatic mitochondria attenuate western diet-induced diabetes. Nat Commun. (2021) 12:101. doi: 10.1038/s41467-020-20313-x, PMID: 33397942 PMC7782853

[50] Chambers ES Viardot A Psichas A Morrison DJ Murphy KG Zac-Varghese SE . Effects of targeted delivery of propionate to the human colon on appetite regulation, body weight maintenance and adiposity in overweight adults. Gut. (2015) 64:1744–54. doi: 10.1136/gutjnl-2014-307913, PMID: 25500202 PMC4680171

[51] Zeng X Chen L Zheng B. Extrusion and chlorogenic acid treatment increase the ordered structure and resistant starch levels in rice starch with amelioration of gut lipid metabolism in obese rats. Food Funct. (2024) 15:5224–37. doi: 10.1039/d3fo05416k, PMID: 38623646

[52] Chen LH Chen YH Cheng KC Chien TY Chan CH Tsao SP . Antiobesity effect of *Lactobacillus reuteri* 263 associated with energy metabolism remodeling of white adipose tissue in high-energy-diet-fed rats. J Nutr Biochem. (2018) 54:87–94. doi: 10.1016/j.jnutbio.2017.11.004, PMID: 29329013

[53] Liu C Du MX Xie LS Wang WZ Chen BS Yun CY . Gut commensal *Christensenella minuta* modulates host metabolism via acylated secondary bile acids. Nat Microbiol. (2024) 9:434–50. doi: 10.1038/s41564-023-01570-0, PMID: 38233647

[54] Lau E Carvalho D Freitas P. Gut microbiota: association with NAFLD and metabolic disturbances. Biomed Res Int. (2015) 2015:979515. doi: 10.1155/2015/979515, PMID: 26090468 PMC4452311

[55] Jacob T Sindhu S Hasan A Malik MZ Arefanian H Al-Rashed F . Soybean oil-based HFD induces gut dysbiosis that leads to steatosis, hepatic inflammation and insulin resistance in mice. Front Microbiol. (2024) 15:1407258. doi: 10.3389/fmicb.2024.1407258, PMID: 39165573 PMC11334085

[56] Natividad JM Lamas B Pham HP Michel ML Rainteau D Bridonneau C . *Bilophila wadsworthia* aggravates high fat diet induced metabolic dysfunctions in mice. Nat Commun. (2018) 9:2802. doi: 10.1038/s41467-018-05249-7, PMID: 30022049 PMC6052103

[57] Lin YC Lin HF Wu CC Chen CL Ni YH. Pathogenic effects of Desulfovibrio in the gut on fatty liver in diet-induced obese mice and children with obesity. J Gastroenterol. (2022) 57:913–25. doi: 10.1007/s00535-022-01909-0, PMID: 35976494

[58] Fan Y Pedersen O. Gut microbiota in human metabolic health and disease. Nat Rev Microbiol. (2021) 19:55–71. doi: 10.1038/s41579-020-0433-9, PMID: 32887946

[59] Ley RE Turnbaugh PJ Klein S Gordon JI. Microbial ecology: human gut microbes associated with obesity. Nature. (2006) 444:1022–3. doi: 10.1038/4441022a 17183309

[60] Qiao S Liu C Sun L Wang T Dai H Wang K . Gut *Parabacteroides merdae* protects against cardiovascular damage by enhancing branched-chain amino acid catabolism. Nat Metab. (2022) 4:1271–86. doi: 10.1038/s42255-022-00649-y, PMID: 36253620

[61] Wang T Han J Dai H Sun J Ren J Wang W . Polysaccharides from Lyophyllum decastes reduce obesity by altering gut microbiota and increasing energy expenditure. Carbohydr Polym. (2022) 295:119862. doi: 10.1016/j.carbpol.2022.119862, PMID: 35989006

[62] Koh A De Vadder F Kovatcheva-Datchary P Backhed F. From dietary fiber to host physiology: Short-chain fatty acids as key bacterial metabolites. Cell. (2016) 165:1332–45. doi: 10.1016/j.cell.2016.05.041, PMID: 27259147

[63] Frost G Sleeth M Sahuri-Arisoylu M Sleeth ML Lizarbe B Cerdan S . The short-chain fatty acid acetate reduces appetite via a central homeostatic mechanism. Nat Commun. (2014) 5:3611. doi: 10.1038/ncomms4611, PMID: 24781306 PMC4015327

[64] Kondo T Kishi M Fushimi T Ugajin S Kaga T. Vinegar intake reduces body weight, body fat mass, and serum triglyceride levels in obese Japanese subjects. Biosci Biotechnol Biochem. (2009) 73:1837–43. doi: 10.1271/bbb.90231, PMID: 19661687

[65] Wu W Chen Z Han J Qian L Wang W Lei J . Endocrine, genetic, and microbiome nexus of obesity and potential role of postbiotics: a narrative review. Eat Weight Disord. (2023) 28:84. doi: 10.1007/s40519-023-01593-w, PMID: 37861729 PMC10589153

[66] Prabhu R Altman E Eiteman MA. Lactate and acrylate metabolism by *Megasphaera elsdenii* under batch and steady-state conditions. Appl Environ Microbiol. (2012) 78:8564–70. doi: 10.1128/AEM.02443-12, PMID: 23023753 PMC3502912

[67] Li N Niu L Liu Y Wang Y Su X Xu C . Taking SCFAs produced by *Lactobacillus reuteri* orally reshapes gut microbiota and elicits antitumor responses. J Nanobiotechnol. (2024) 22:241. doi: 10.1186/s12951-024-02506-4, PMID: 38735933 PMC11089779

[68] Falcinelli S Rodiles A Hatef A Picchietti S Cossignani L Merrifield DL . Influence of probiotics administration on gut microbiota core: a review on the effects on appetite control, glucose, and lipid metabolism. J Clin Gastroenterol. (2018) 52:S50–6. doi: 10.1097/MCG.0000000000001064, PMID: 29864068

[69] Vitetta L Briskey D Alford H Hall S Coulson S. Probiotics, prebiotics and the gastrointestinal tract in health and disease. Inflammopharmacology. (2014) 22:135–54. doi: 10.1007/s10787-014-0201-4, PMID: 24633989

[70] Liu R Hong J Xu X Feng Q Zhang D Gu Y . Gut microbiome and serum metabolome alterations in obesity and after weight-loss intervention. Nat Med. (2017) 23:859–68. doi: 10.1038/nm.4358, PMID: 28628112

[71] Ryu SW Moon JC Oh BS Yu SY Bak JE Heo ES . Anti-obesity activity of human gut microbiota *Bacteroides stercoris* KGMB02265. Arch Microbiol. (2023) 206:19. doi: 10.1007/s00203-023-03750-2, PMID: 38086977

[72] Lopez-Almela I Romani-Perez M Bullich-Vilarrubias C Benitez-Paez A Gomez Del Pulgar EM Frances R . *Bacteroides uniformis* combined with fiber amplifies metabolic and immune benefits in obese mice. Gut Microbes. (2021) 13:1–20. doi: 10.1080/19490976.2020.1865706, PMID: 33499721 PMC8018257

[73] Nagpal R Wang S Solberg Woods LC Seshie O Chung ST Shively CA . Comparative microbiome signatures and Short-chain fatty acids in mouse, rat, non-human primate, and human feces. Front Microbiol. (2018) 9:2897. doi: 10.3389/fmicb.2018.02897, PMID: 30555441 PMC6283898

[74] Walter J Ley R. The human gut microbiome: ecology and recent evolutionary changes. Ann Rev Microbiol. (2011) 65:411–29. doi: 10.1146/annurev-micro-090110-102830, PMID: 21682646

[75] Gaulke CA Sharpton TJ. The influence of ethnicity and geography on human gut microbiome composition. Nat Med. (2018) 24:1495–6. doi: 10.1038/s41591-018-0210-8, PMID: 30275567

[76] Chernonosov AA Mednova IA Levchuk LA Mazurenko EO Roschina OV Simutkin GG . Untargeted plasma metabolomic profiling in patients with depressive disorders: a preliminary study. Meta. (2024) 14:110. doi: 10.3390/metabo14020110, PMID: 38393002 PMC10890195

[77] Plovier H Everard A Druart C Depommier C Van Hul M Geurts L . A purified membrane protein from Akkermansia muciniphila or the pasteurized bacterium improves metabolism in obese and diabetic mice. Nat. Med. (2017) 23:107–113. doi: 10.1038/nm.4236, PMID: 27892954

