# Supplementary material for: Christensenella intestinihominis MNO-863 improve obesity and related metabolic disorders via SCFAs-induced GLP-1 hormone secretion
Source: Front Nutr. 2025 Nov 20;12:1668786. doi: 10.3389/fnut.2025.1668786 (PMC12677068; doi:10.3389/fnut.2025.1668786)
Supplement: Supplementary file 1 [file Table_1.DOCX]

Supplementary Materials for

***Christensenella intestinihominis* MNO-863 improve Obesity and Related Metabolic Disorders via** **SCFAs-induced GLP-1 hormone secretion**

**Authors and Affiliations**

Ping Kong^1，#^, Yibo Xian^1，#^, Canshan Lao^1^, Baojia Huang^1^, Dongya Zhang^1^, Lihong Tai^1^, Yingying Zhao^1^, Zilun Pu^1^, Zhou Lan^1^, Chenchen Zhang^1^, Zhenzhen Liu^1^, Chen Xiao^1^, Guozhen Zhao^1^, Ruijuan Zhu^1^, Yajun Liang^1^, Chuan-Sheng Lin^1^, Jing-han Lin^2^, Jing-zu Sun ^2^, Tao Wang^2^, Hong-Wei Liu^2^*, Xianzhi Jiang^1^*

1. Moon (Guangzhou) Biotech Co. Ltd., Huangpu District, Guangzhou, Guangdong, 510530, China

2. State Key Laboratory of Microbial Diversity and Innovative Utilization, Institute of Microbiology, Chinese Academy of Sciences, Beijing, P. R. China.

^#^ These authors contributed equally: Ping Kong and Yibo Xian

*Corresponding author: Xianzhi Jiang, Email: [**jxz@moonbio.com**](mailto:jxz@moonbio.com); Fax: 020-31603387; Telephone: +86-13681189946; Hong-Wei Liu, Email: [**liuhw@im.ac.cn**](mailto:liuhw@im.ac.cn)

**Materials and Methods**

**Genome sequencing and analysis**

Bacterial DNA of Strains from MoonBiotech was extracted by Findrop Magnetic Bead Extraction Kit (Fangzhou Biosafety Technology (Guangzhou) Co., LTD) according to manufacturer’s instruction. DNA library was constructed using the VAHTS Universal DNA Library Prep Kit for Illumina V3 (Novizan Biotechnology Co., LTD., China) for IlluminaV3 following the manufacturer’s instruction, and whole-genome shotgun sequenced using the NextSeq 2000 instrument, which generated a series of 150 bp paired-end reads. Reads of each strain were then filtered with fastp (version 0.20.0) and assembled with SPAdes (version 3.14.0). Strain species was identified with GTDB-Tk (version 2.3.2) based on assembly.

Cohorts’ data were retrieved from public databases following project numbers PRJEB12123 (obesity, n = 104), PRJEB21528(cardiovascular disease and health, n = 405 (218, 187)), PRJNA422434 (type 2 diabetes and health, n = 359 (187, 172)), PRJNA373901(nonalcoholic fatty liver disease and health, n = 86 (50,36)), PRJNA278393 (health, n = 38), PRJNA388263 (health, n = 293), respectively. The cohorts and metagenomic data were analyzed using fastp (version 0.24.0) Kraken2(version 2.1.3), Bracken (version 2.9) and R scripts. For analysis of the frequency of occurrence (FO) and relative abundance (RA) of Christensenella strain, a self-built library was generated by including the ChrisGMB strain (n = 68) and *Christensenella* strain from MoonBiotech (n = 206) into Kraken2 standard database.

To identify and compare the functional categories of *C. intestinihominis* and *C. minuta*, Clusters of Orthologous Groups (COGs) databases were used. Protein sequences produced by PROKKA from strain assembly were aligned to COG sequences by DIAMOND (version 0.9.24.125).

**Toxicity study**

The toxicity study was conducted in accordance with the National Medical Products Administration (NMPA) GLP Regulations (No.34, 2017) and the United States Food and Drug Administration (FDA) Good Laboratory Practice (GLP) Regulations (21 CFR Part 58) in the Medicilon Preclinical Research (Shanghai) LLC. The test articles were MNO-863 Fermental Powder (MNO-863 and protective agent lyophilized powder) and MNO-863 Protective Agent Lyophilized Powder (Negative control) in the toxicity study. Based on the stability data of the microbial powder, the Fermental Powder with 1×10^12^CFU/g can be stored stably at 2-8℃ for 2 years. After being formulated into a bacterial suspension for administration, it can be stored stably at 2-8℃ for 7 days. The results of Formulation analysis, including the number of live bacteria and the homogeneity, were accurate.

The rat is selected because it is a rodent species and commonly used in toxicology studies, and with extensive historical control data. The oral administration selected is because it is an intended clinical route.

One hundred and sixty SD rats (SPF grade, 80 males and 80 females) were included in this study. They were randomly divided into 8 groups according to gender and body weight. Animals in Groups 1-4 were used for the toxicity study (15 animals per sex in each group). Animals in Groups 5-8 were used for the toxicokinetic study (5 animals per sex in Groups 5-8, which 1 animal per sex in each group as back-up animals). Animals in Groups 1 and 5 were dosed with the negative control (MNO-863 Protective Agent Lyophilized Powder, 0 CFU/Animal/Day), and animals in Groups 2 to 4 and Groups 6 to 8 were dosed with MNO-863 Fermental Powder at 1.2×10^11^, 6×10^11^ and 1.2×10^12^ CFU/Animal/Day, respectively. Animals in all groups were administered twice daily via oral gavage with the dose volume of 6mL/Animal/Day. Justification of Dose Levels: Given that the preclinical effective dose of anti-obesity effect of MNO-863 is 1.2×10^11^ CFU/Animal/Day, the dosage selected here for group 2 (low), group 3 (medium) and group 4 (high) is 1 time, 5 times, and 10 times of the preclinical effective dose, respectively. Therefore, in this study, the low, medium and high doses were set at 1.2×10^11^, 6×10^11^ and 1.2×10^12^ CFU/Animal/Day respectively.

The treatment had lasted for 28 days following a 28 days-recovery. Body weight and food consumption were measured during the study. Clinical pathology examinations including hematology, coagulation, clinical chemistry and urinalysis were conducted at the end of the dosing period (Day 29) and at the end of the recovery period (Day 57). After overnight fasting on Days 29 and 57, the rats were given 25 mg/kg Zoletil 50 (Virbac, 9RVSB) intramuscularly. Once deep anesthesia was confirmed, the abdominal aorta was cannulated for terminal blood collection and immediate exsanguination, ensuring rapid death. Necropsy was conducted after the blood collection of clinical pathology. The corresponding tissues were collected, weighed, preserved and microscopically examined. After the experiment, the data process and statistical analysis of the corresponding test indicators were performed based on the comparison of the test article treatments with the negative control. For groups 1-4, 10 animals/sex/group were necropsied at the end of the dosing phase (Day 29), and 5 animals/sex/group were necropsied at the end of the recovery period (Day 57). Organ weights, and gross/microscopic observations were accessed and evaluated. For the toxicokinetic study, the time points of blood collection from animals in the Group 5-8 were at pre-dose, 1, 3, 6, 7, 9 and 24 h post dosing after the first administration on Day 1 and Day 28, respectively.

Supplementary figures


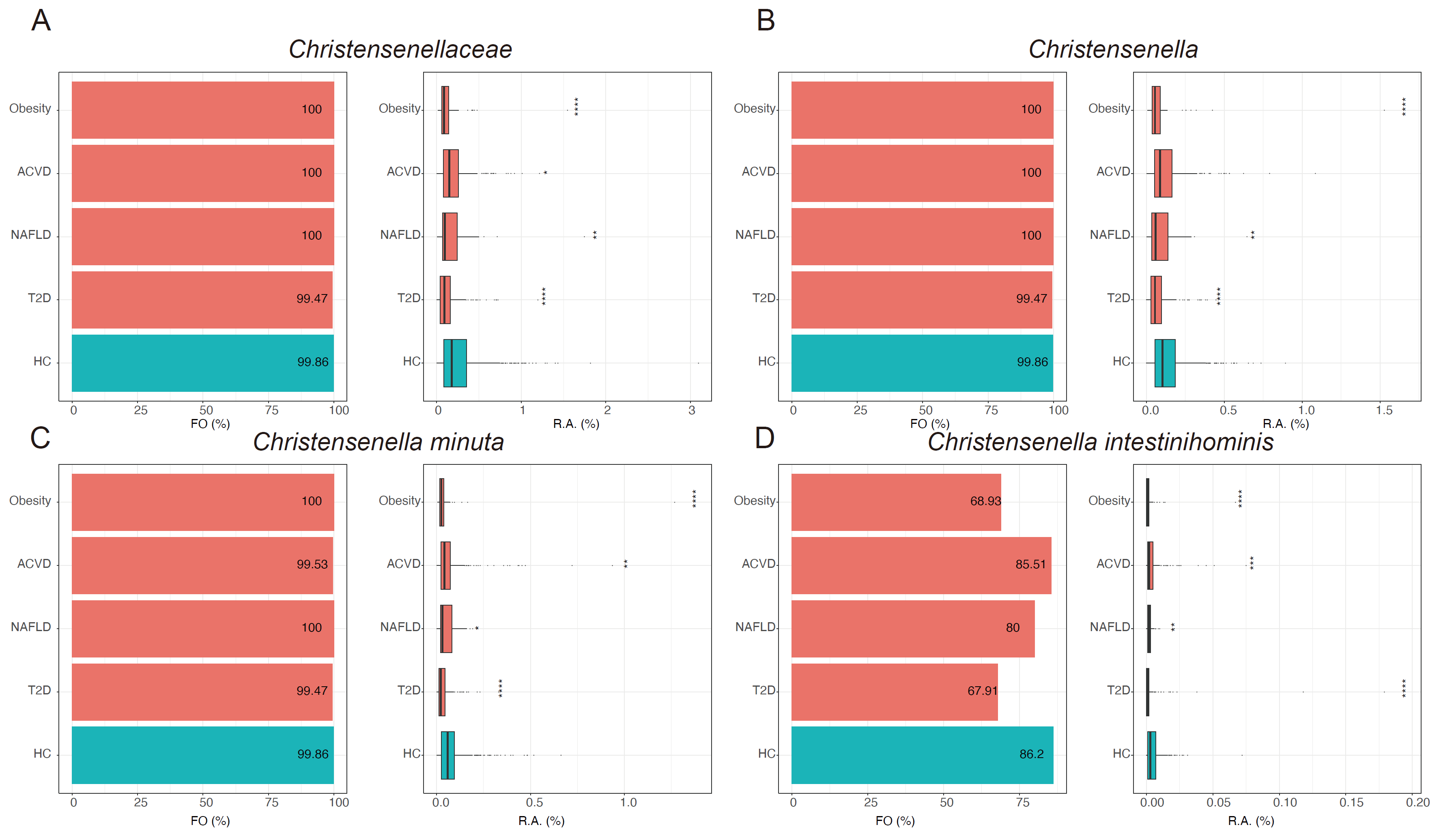


Fig. S1. *Christensenellaceae, Christensenella*, *Christensenella minuta* and *Christensenella intestinihominis* were significantly reduced in patients with obesity (OB), nonalcoholic fatty liver disease (NAFLD), type 2 diabetes (T2D) and Atherosclerotic cardiovascular disease (ACVD). A non-parametric Kruskal–Wallis test followed by Dunn’s multiple comparisons test was conducted for statistical analysis. **p* < 0.05, ***p* < 0.01, ****p* < 0.001, *****p* < 0.0001.


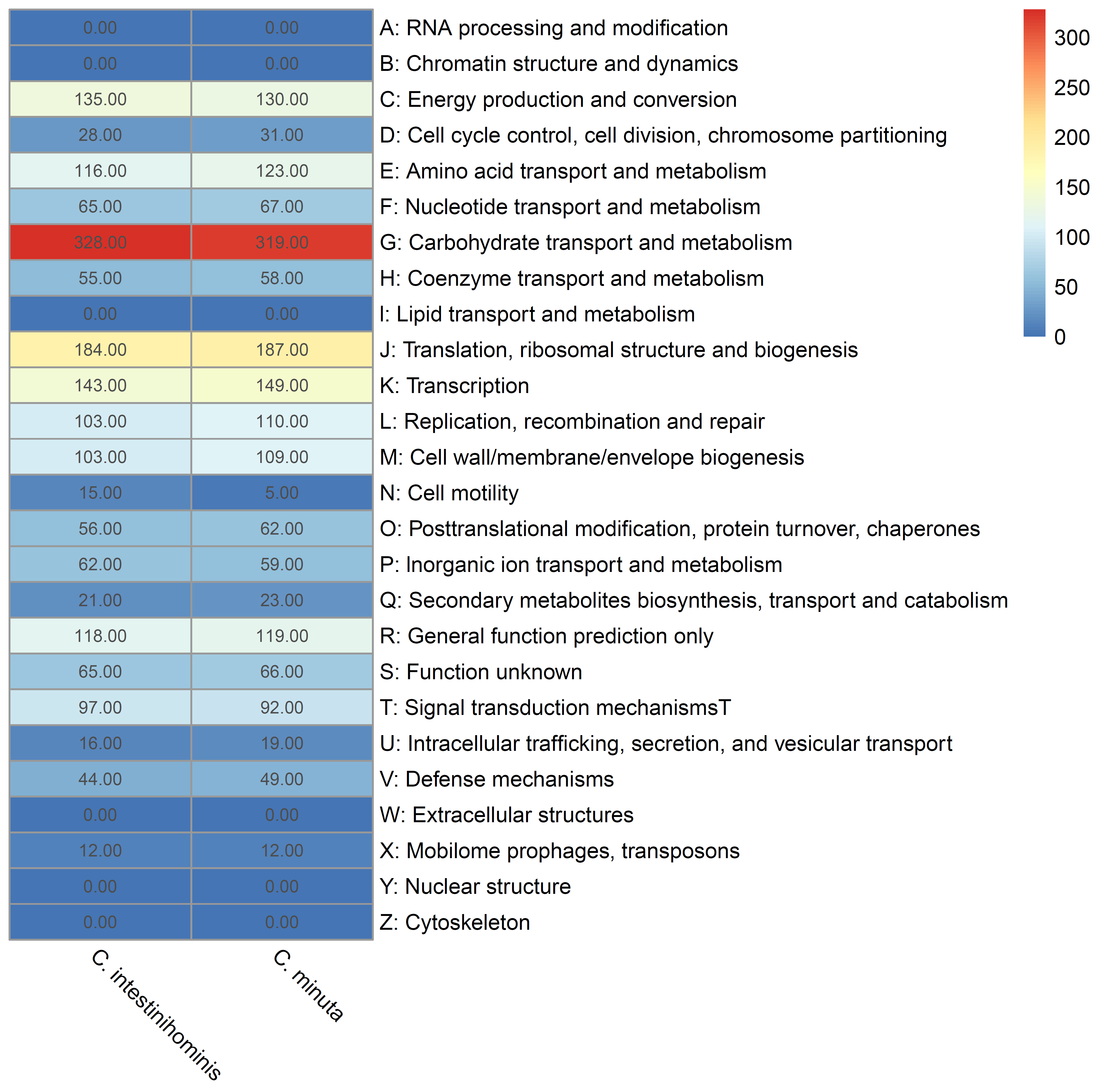


Fig. S2. The comparison results of COG category average number between twelve *C. intestinihominis* strains and eighty-three *C. minuta* strains.

Fig. S3. Effects of 14 strains, including 4 *Christensenella* strains and 10 strains of other 9 species that have previously reported weight loss effects (*Akkermansia muciniphila*, *Akkermansia massiliensis*, *Bacteroides thetaiotaomicron*, *Parabacteroides distasonis*, *Intestinimonas butyricciproducens*, *Parabacteroides goldsteinii*, *Coprococcus comes*, *Blautia obeum* and *Blautia wexlerae*) on body weight in HFD-induced obese mice. Data are presented as Mean ± SD. Statistical analysis was performed by one-way ANOVA combined with Dunnett’s multiple comparisons test. *: *p* < 0.05 and **: *p* < 0.01.


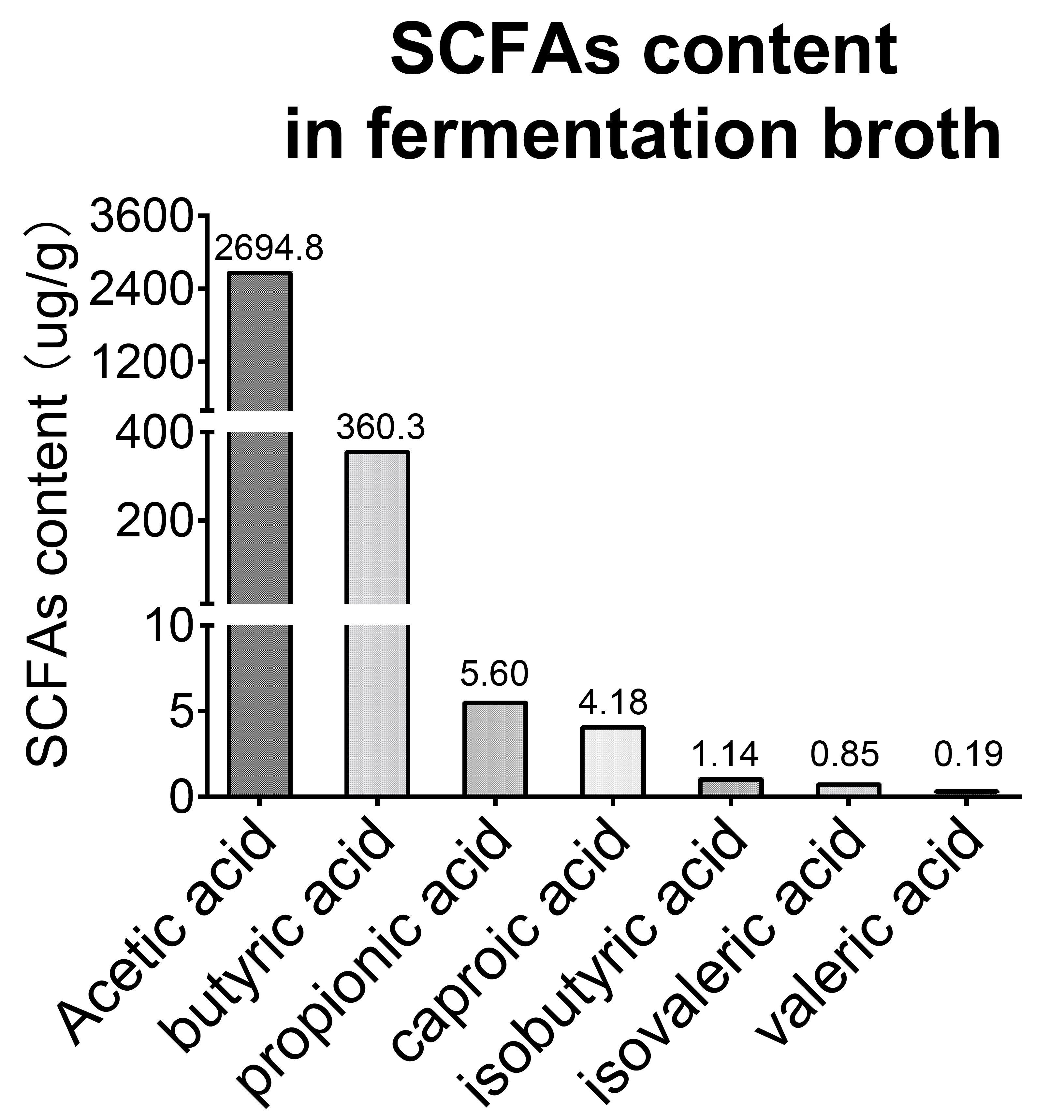


Fig. S**4**. SCFA content in MNO-863 fermentation broth.





Fig. S5. Effects of MNO-863 Fermentation Powder on body weight and food consumption of male and female animals in each group during the dosing and the recovery period.

(A) Curve graph of body weight in male animals. (B) Curve graph of body weight in female animals. (C) Food consumption in male animals. (D) Food consumption in female animals.

**Table S1 Summary data of plasma hormones.**

| Indicators | HFD-Vehicle | MNO-863 | |
| --- | --- | --- | --- |
|  | Mean±SD | Mean±SD | %Diff |
| GLP-1 (active) (pg/mL) | 23.153±3.195 | 62.791±30.519 | 171.2** |
| GIP (total) (pg/mL) | 409.328±119.507 | 122.042±39.306 | -70.2**** |
| PYY (pg/mL) | 62.356±27.550 | 166.560±62.797 | 167.1*** |
| Secretin (pg/mL) | 19.678±7.642 | 44.804±17.002 | 127.7*** |
| Resistin (pg/mL) | 21221.222±2950.863 | 13457.778±3419.294 | -36.6**** |
| Leptin (pg/mL) | 43244.000±12414.279 | 24536.444±13332.603 | -43.3** |
| Amylin (active) (pg/mL) | 73.350±31.344 | 75.877±40.921 | 3.4 |
| Ghrelin (active) (pg/mL) | 170.412±139.909 | 306.942±162.404 | 80.1 |
| C-Peptide 2 (pg/mL) | 1643.444±454.428 | 1289.809±343.769 | -21.5 |
| Insulin (pg/mL) | 2574.850±1157.876 | 2033.222±580.546 | -21.0 |
| Glucagon (pg/mL) | 77.166±33.636 | 71.166±36.647 | -7.8 |
| IL-6 (pg/mL) | 26.888±10.454 | 29.598±15.754 | 10.1 |
| TNF-α (pg/mL) | 6.728±1.759 | 9.206±2.084 | 36.8 |
| LPS (ng/mL) | 2.481±0.371 | 1.977±0.270 | -20.3** |

All data are expressed as Mean±SD. %Diff = (mean value of MNO-863 group - mean value of HFD-Vehicle group)/mean value of HFD-Vehicle group×100%. Statistical analysis using GraphPad software (version 10.2.3) was performed by two-tail unpaired Student’s t test. ns: not significant, not showed; **: *p* < 0.01, ***: *p* < 0.001 and ****: *p* < 0.0001.

Table S2 Summary data of relative concentration for obesity-related factors.

| Indicators | HFD-Vehicle | MNO-863 | |
| --- | --- | --- | --- |
|  | Mean±SD | Mean±SD | %Diff |
| CCL2/JE/MCP-1 | 1±0.093 | 0.942±0.064 | -5.8 |
| DPPIV/CD26 | 1±0.180 | 0.659±0.111 | -34.1** |
| IGFBP-1 | 1±0.183 | 1.454±0.375 | 45.4 |
| ICAM-1/CD54 | 1±0.201 | 1.313±0.458 | 31.3 |
| Resistin | 1±0.104 | 0.957±0.112 | -4.3 |
| CCL5/RANTES | 1±0.293 | 0.752±0.063 | -24.8 |
| Serpin E1/PAI-1 | 1±0.231 | 0.702±0.223 | -29.8* |
| Prolactin | 1±0.391 | 1.005±0.420 | 0.5 |
| M-CSF | 1±0.142 | 1.088±0.178 | 8.8 |
| Proprotein Convertase 9/PCSK9 | 1±0.661 | 1.07±0.097 | 7.0 |
| BAFF/BLyS/TNFSF13B | 1±0.492 | 0.975±0.425 | -2.5 |
| FGF-21 | 1±0.817 | 0.666±0.220 | -33.4 |
| C-Reactive Protein/CRP | 1±0.141 | 0.848±0.041 | -15.2* |
| FGF basic/FGF2/bFGF | 1±0.170 | 1.016±0.186 | 1.6 |
| Angiopoietin-2 | 1±0.240 | 0.912±0.085 | -8.8 |
| Complement Factor D/Adipsin | 1±0.209 | 1.211±0.234 | 21.1 |
| Oncostatin M/OSM | 1±0.246 | 1.193±0.180 | 19.3 |

All data are relative concentration (obtained as a relative value by normalizing with HFD-Vehicle) and expressed as Mean±SD. %Diff = (mean value of MNO-863 group - mean value of HFD-Vehicle group)/mean value of HFD-Vehicle group×100%. Statistical analysis using GraphPad software (version 10.2.3) was performed by two-tail unpaired Student’s t test. ns: not significant, not showed; **p*＜0.05, **: *p* < 0.01.

**Table S3 Statistically significant changes of clinical pathology compared with concurrent negative control group.**

| Clinical pathology | Index | Sex | Group | %Diff | Main judgment basis |
| --- | --- | --- | --- | --- | --- |
| Hematology | EOS | Male^a^ | 2 | ↓52.68%^**^ | Within the normal range |
|  | BASO | Male^a^ | 2 | ↓65.38%^*^ | No dose relationship and within the normal range |
|  |  |  | 4 | ↓76.92%^**^ |  |
|  |  | Female^a^ | 4 | ↓72.22%^*^ | Within the normal range |
|  | BASO% | Male^a^ | 2 | ↓58.33%^*^ | No dose relationship and within the normal range |
|  |  |  | 4 | ↓66.67%^*^ |  |
|  |  | Female^a^ | 4 | ↓72.73%^*^ | Within the normal range |
|  | RET | Male^a^ | 2 | ↓14.62%^**^ | Within the normal range |
|  | MONO | Female^a^ | 4 | ↓35.59%^*^ | Within the normal range |
| Coagulation | APTT | Male^a^ | 2 | ↑11.24%^**^ | No dose relationship |
|  |  |  | 4 | ↑8.68%^*^ |  |
|  |  | Female^a^ | 4 | ↑12.92%^**^ | Within the normal range |
|  | FBG | Male^b^ | 4 | ↓10.30%^*^ | Within the normal range |
| Clinical chemistry | TCHO | Male^a^ | 2 | ↓19.5%^*^ | Within the normal range |
|  | ALB | Female^a^ | 4 | ↓8.33%^**^ | Within the normal range |
|  |  | Male^b^ | 3 | ↓5.72%^*^ | No dose relationship and within the normal range |
|  |  |  | 4 | ↓4.60%^*^ |  |
|  | TBIL | Female^a^ | 2 | ↓15.24%^*^ | No dose relationship and within the normal range |
|  |  |  | 4 | ↓13.00%^*^ |  |
|  | TP | Female^a^ | 4 | ↓7.39%^**^ | Small change and within the normal range |
|  |  | Male^b^ | 4 | ↓6.33%^*^ |  |
|  | CRE | Male^b^ | 4 | ↓10.24%^*^ | Within the normal range |
|  | GLO | Male^b^ | 4 | ↓8.03%^*^ | Within the normal range |
|  | TG | Female^b^ | 4 | ↑25.2%^**^ | Within the normal range |
| Urinalysis | SG | Female^a^ | 3 | ↑0.88%^**^ | Within the normal range and No dose correlation and |
|  | Volume | Female^a^ | 3 | ↓39.67%^*^ |  |

Note: %Diff = (mean value of test article group - mean value of concurrent negative control group)/mean value of concurrent negative control group×100%; ↓: decrease, ↑: increase; If the sample size of each group is not less than 3, Levene's test is used to perform homogeneity statistics for the specific end point data (refer to the above table) and the number of collection periods. If there was no significant difference in the results of Levene's test (*p* ≥0.01), one-way analysis of variance (mean square error) was performed using ANOVA, and Dunnett's test was used to compare the administered and control groups. If there were significant differences in the results of Levene's test (*p* < 0.01), Welch+Bonferroni test was used to compare the treatment group and control group. **p*＜0.05, ***p*＜0.01.

a, represent the end of the dosing period (Day 29);

b, represent the end of the recovery period (Day 57).

**Table S4 Statistically significant changes of organ weight compared with concurrent negative control group.**

| Index | Sex | Group | %Diff | Main judgment basis |
| --- | --- | --- | --- | --- |
| Kidneys/TBWT | Female^a^ | 3 | ↑9.15427%^*^ | No difference in absolute weight with no abnormalities in histopathology |
| Brain/TBWT | Male^b^ | 3 | ↑14.1026%^**^ | No dose relationship |
| Spleen/TBWT | Female^b^ | 4 | ↓24.72956%^**^ | No difference in absolute weight with no abnormalities in histopathology |

Note: %Diff = (mean value of test article group - mean value of concurrent negative control group)/mean value of concurrent negative control group×100%; ↓: decrease, ↑: increase. If the sample size of each group is not less than 3, Levene's test is used to perform homogeneity statistics for the specific end point data (refer to the above table) and the number of collection periods. If there was no significant difference in the results of Levene's test (*p* ≥0.01), one-way analysis of variance (mean square error) was performed using ANOVA, and Dunnett's test was used to compare the administered and control groups. If there were significant differences in the results of Levene's test (*p* < 0.01), Welch+Bonferroni test was used to compare the treatment group and control group. **p*＜0.05, ***p*＜0.01.

a, represent the end of the dosing period (Day 29);

b, represent the end of the recovery period (Day 57).
